# Everything you ever wanted to know about the Think/No-Think task, but forgot to ask

**DOI:** 10.3758/s13428-024-02349-9

**Published:** 2024-02-20

**Authors:** Davide Nardo, Michael C. Anderson

**Affiliations:** 1 Department of Education, University of Roma Tre, Rome, Italy; 2 MRC Cognition and Brain Sciences Unit, University of Cambridge, Cambridge, UK

**Keywords:** Think/No-Think task, Episodic memory, Memory control, Retrieval suppression, Suppression-induced forgetting, Motivated forgetting, Replicability, Training materials, Intrusive thoughts

## Abstract

The Think/No-Think (TNT) task has just celebrated 20 years since its inception, and its use has been growing as a tool to investigate the mechanisms underlying memory control and its neural underpinnings. Here, we present a theoretical and practical guide for designing, implementing, and running TNT studies. For this purpose, we provide a step-by-step description of the structure of the TNT task, methodological choices that can be made, parameters that can be chosen, instruments available, aspects to be aware of, systematic information about how to run a study and analyze the data. Importantly, we provide a TNT training package (as Supplementary Material), that is, a series of multimedia materials (e.g., tutorial videos, informative HTML pages, MATLAB code to run experiments, questionnaires, scoring sheets, etc.) to complement this method paper and facilitate a deeper understanding of the TNT task, its rationale, and how to set it up in practice. Given the recent discussion about the replication crisis in the behavioral sciences, we hope that this contribution will increase standardization, reliability, and replicability across laboratories.

## Background

### Theoretical framework

#### Introduction

Everyone has been reminded of an unwelcome past experience that they would rather not think about. Such reminders can arise abruptly and involuntarily: whether we smell our ex-partner’s perfume on another, encounter a lost loved one’s picture, or stumble into a situation resembling a prior embarrassment or trauma, such cues evoke memories that leave distress in their wake. The most common way to prevent such unpleasant experiences is to avoid a given reminder entirely. However, sometimes we cannot avoid unwelcome reminders. When this happens, we often attempt to exclude the unwanted memory from our awareness. In doing so, we exert a type of cognitive control called “retrieval suppression”, that is, we actively stop the memory retrieval process. Indeed, in everyday life we resort to retrieval suppression more often than we realize. It has been suggested that retrieval suppression plays a positive, protective role in maintaining mental health (Engen & Anderson, 2018; Mamat & Anderson, 2023), and evidence suggests that many psychological disorders are associated with diminished memory control capability (Stramaccia et al., 2021; Pevie et al., 2023; Section Clinical populations).

In two decades, suppression-induced forgetting (Section Suppression-Induced Forgetting (SIF)) has been investigated using the Think/No-Think (hereinafter, “TNT”) task (Anderson & Green, 2001) as a means of inducing retrieval suppression. A growing number of researchers have used this task and adapted it to differing research needs, including behavioral and neuroimaging studies, healthy and clinical and neuropsychological cohorts, children, adults, and aging populations. Briefly (a detailed protocol is provided in Section TNT task structure, explaining its structure in a step-by-step manner), in a TNT task participants: i) learn associations between pairs of items (e.g., words or pictures); ii) repeatedly retrieve or suppress the second pair member (target) when shown the first member (cue); and iii) recall all learned pairs, i.e., recall targets when shown the cues. We have collaborated with many research groups worldwide, sharing our experience and practical knowledge about this protocol. Despite this, measuring suppression-induced forgetting using the TNT paradigm poses methodological challenges that are best addressed through careful training (Section Training researchers). Therefore, we sought here to standardize training and methods for studies using the TNT task. We aim to increase the reliability, replicability (more about this in Section Replicability of the Suppression-Induced Forgetting effect), and interpretability of research on retrieval suppression, with the broader goal of improving the quality, amount, and diversity of the science done using this method. We start with a brief theoretical overview and history to illustrate how work on retrieval suppression has evolved over two decades.

#### An active mechanism of forgetting

**Fig. 1:**
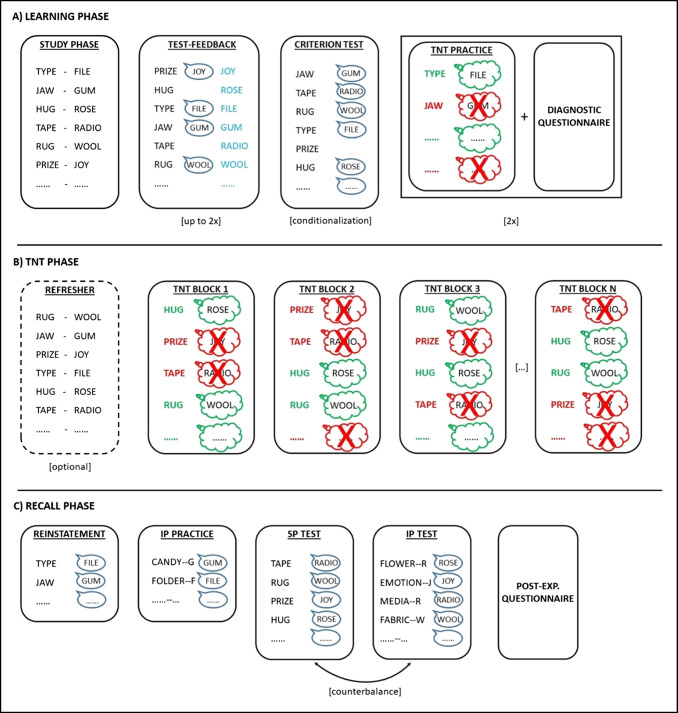
Typical TNT task structure. **A) **The learning phase is made up of: study phase, test-feedback phase (which may be repeated up to two times), criterion test (whose score is used for conditionalization; Section Conditionalized data), TNT practice (performed twice), each of which is followed by the administration of the diagnostic questionnaire (Section Diagnostic questionnaire (DQ)). **B)** The TNT phase is made up of: an optional pair refresher (only when needed), a series of TNT blocks (typically 4–5) interspersed with short breaks. **C)** The Recall phase is made up of: study-context reinstatement, independent-probe (IP) practice, same-probe (SP) test and IP test (whose order is counterbalanced across subjects), followed by the post-experimental questionnaire (Section Post-experimental questionnaire (PEQ))

**Fig. 2:**
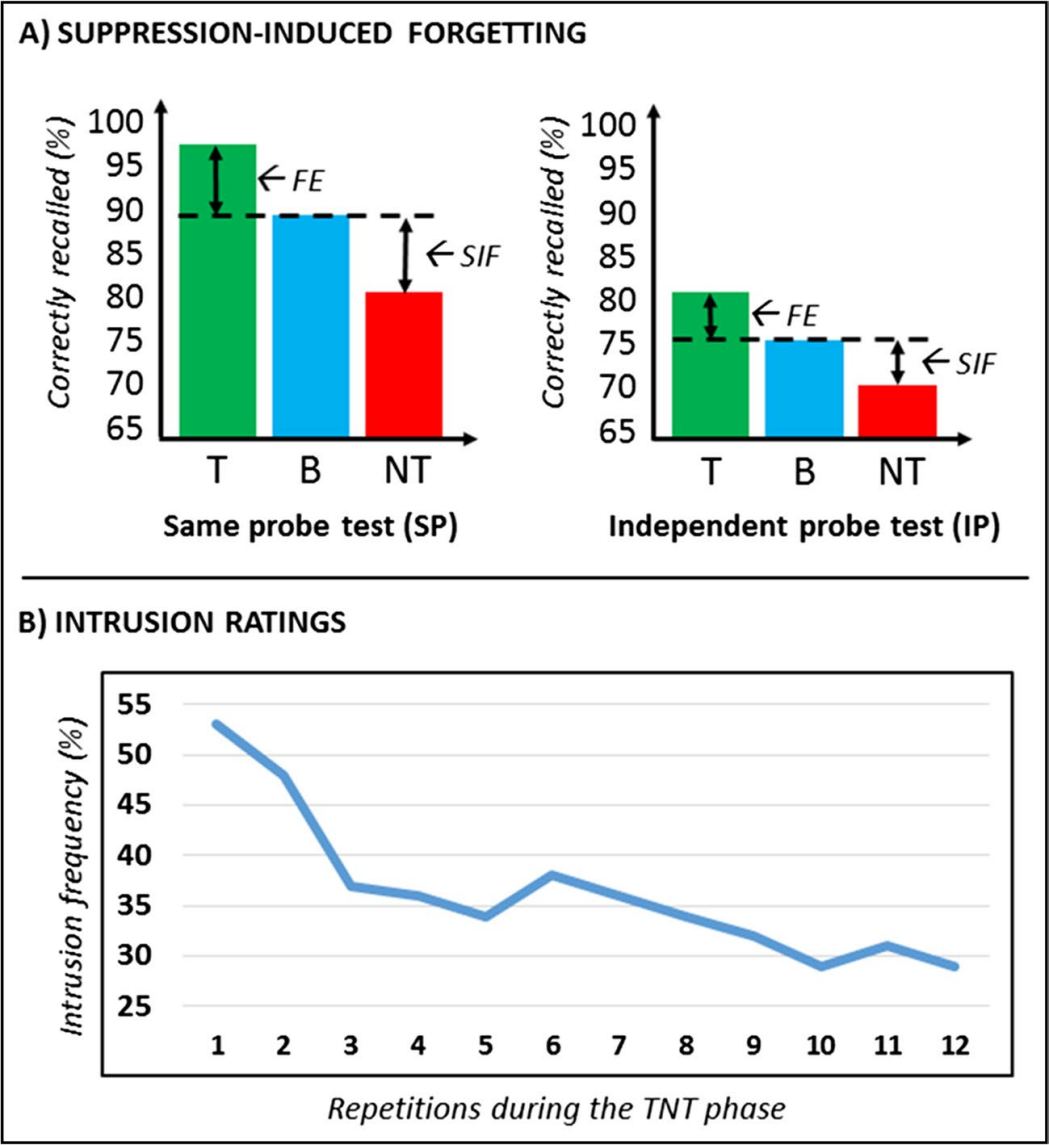
Typical behavioral results obtained in a TNT study. **A)** Suppression-induced forgetting effect (SIF; Section Introduction, Suppression-Induced Forgetting (SIF), Suppression ANOVA), and facilitatory effect (FA; Section Facilitation ANOVA) based on performance at the recall phase, separately for same probe (SP) and independent probe (IP) tests. The figures portrayed are based on 32 published TNT studies (cf. Anderson & Huddleston, 2012). **B)** Trend of intrusion ratings (Sections Intrusion ratings (theory), Intrusion frequency, Intrusion slope) as a function of the number of repetitions (i.e., suppressions of NT trials) during the TNT phase, showing the characteristic decline over repetitions. The figures portrayed are based on the data of 48 participants (cf. Levy & Anderson, 2012)

We introduced the TNT task to investigate the role of retrieval suppression in active forgetting (Anderson & Green, 2001). To address this issue, our TNT task modifies the Go/No-Go task^1^ typically used in studies on motor inhibition to investigate the stopping of internal actions like episodic memory retrieval (see Section TNT task structure for a full description of the TNT task and its phases, and Fig. 1 for a schematic overview). Our findings revealed that suppressing the retrieval of a memory impaired its later retention, a phenomenon we refer to as “suppression-induced forgetting” (from now on “SIF”; Section Suppression-Induced Forgetting (SIF); cf. Fig. 2A). SIF increases (although non-linearly) with the number of times that retrieval is stopped, is resistant to incentives for accurate recall, and broadly disrupts retention of the suppressed content. Importantly, our evidence indicates that retrieval suppression arises, in part, from inhibitory control mechanisms acting on the suppressed memory and not simply associative interference^2^ (Anderson & Spellman, 1995; Anderson & Neely, 1996; Wang et al., 2015; see Section Independent-probe (IP) test for further details about these mechanisms). We have argued that such inhibitory mechanisms provide a psychological model of motivated forgetting (Anderson & Huddleston, 2012).

#### Neural correlates of retrieval suppression

Shortly after developing the TNT task, we used it in a functional MRI study investigating the neural mechanisms of retrieval suppression (Anderson et al., 2004). We found that when contrasting retrieval suppression with active retrieval, areas involved in cognitive control function (notably including bilateral dorsolateral and ventrolateral prefrontal cortices, anterior cingulate cortex, pre-supplementary motor area, and dorsal premotor and posterior parietal cortices) showed significantly increased activity, whereas the hippocampi showed down-regulation (though subsequent meta-analyses have revealed prefrontal activations be strongly right lateralized; e.g., Guo et al. 2018; Apšvalka et al. 2022). Critically, activation in both the dorsolateral prefrontal cortex (DLPFC) and the hippocampus predicted individual differences in the capacity to forget unwanted memories, suggesting that they constitute core regions in a neurobiological model of memory control (Anderson & Hanslmayr, 2014). Later studies showed that suppression of hippocampal processing also induces forgetting of unrelated events experienced before or after periods of suppression, a phenomenon known as “amnesic shadow” (Hulbert et al., 2016). The amnesic shadow suggests that retrieval suppression broadly compromises hippocampal function. This disruption may be linked to the action of GABAergic inhibitory neurons in the hippocampus, which have been shown to play a role in the fronto-hippocampal network underlying memory suppression, such that greater hippocampal GABA concentrations predict superior forgetting, and more robust top-down control by the prefrontal cortex (Schmitz et al., 2017). Through multiple fMRI studies with this method, an increasingly specific neurobiological model of motivated forgetting has emerged (Anderson et al., 2016; Anderson & Hulbert, 2021).

#### Direct suppression vs. thought substitution

At least two mechanisms can be engaged to prevent an unwanted memory from coming to mind, given a reminder: direct suppression and thought substitution (Hertel & Calcaterra, 2005; Bergström et al., 2009; Benoit & Anderson, 2012). Direct suppression refers to the act of stopping the retrieval of an unwanted memory (i.e., “blanking the mind”), either to prevent its content from accessing awareness or to expel it, once in awareness. Thought substitution refers to the exclusion of an unwanted memory from awareness by retrieving an alternative thought or memory (such as another thought, image, or idea) as a way to redirect the mind away from an unwanted content (e.g., thinking about an urgent duty to avoid thinking about an ex-partner). Crucially, despite both mechanisms yielding similar rates of forgetting, they are associated with opposite neural patterns. Whereas direct suppression significantly increases activity in the right DLPFC and down-regulates the hippocampus, thought substitution increases activity in the left ventrolateral prefrontal cortex and sustained or increased activity in the hippocampus (Benoit & Anderson, 2012). Direct suppression and thought substitution have been dissociated in numerous ways, including using EEG, fMRI, and behavioral methods (for a review of these dissociations, see Anderson & Hulbert, 2021).

#### Intrusion ratings (theory)

We introduced intrusion ratings as a way to track phenomenological experiences of memories intruding into awareness during the TNT task (Levy & Anderson, 2012). Subjects are asked after each trial ends, to indicate whether, while seeing the preceding reminder cue, they experienced awareness of the associated target memory by using a three-fold choice: i) never; ii) briefly; and iii) often. Participants are instructed to make this decision quickly (Section TNT phase) without retrieving the associate again; later, if participants respond with answers (ii) and (iii), these responses are collapsed to create a binary score (i.e., “no” vs. “yes”) reflecting whether an intrusion happened or not. This is done because our main concern is a participant’s “reporting bias”. By using the three-ratings option (adopted in most TNT studies tracking intrusions), we aim to capture also potentially subtle intrusions (i.e., we want to make sure that even uncertain, brief, or fuzzy intrusions are fully captured). Then we collapse, as we are interested in distinguishing successfully from non-successfully suppressed trials. Intrusion frequency declines over No-Think repetitions (cf. Fig. 2B), and the rate of this decline (i.e., its slope) often predicts SIF. This decline in intrusions is exceptionally robust and has been confirmed in multiple studies (Levy & Anderson, 2012; Benoit et al., 2015; Hellerstedt et al., 2016; Gagnepain et al., 2017; van Schie & Anderson, 2017; Harrington et al., 2021; Mary et al., 2020; Legrand et al., 2020). Intrusions are associated with greater activity reduction in the hippocampus and more negative top-down coupling, consistent with the possibility that control mechanisms are engaged to purge memories from awareness (Levy & Anderson, 2012; Gagnepain et al., 2017; Mary et al. 2020). Interestingly, DLPFC activation is greatest when unwanted memories intrude into awareness, and its inhibitory influence on the hippocampus is linked to a reduction in intrusions over time (Benoit et al., 2015).

### Development and differentiation of the field

#### Individual differences

To date, the vast majority of TNT studies were run on healthy adults, delineating the typical results reported in the various sections of the present work (cf. Fig. 2). However, there also has been great interest in individual differences in memory suppression. One group of studies has focused on age-related differences. For instance, children exhibit age-related improvements in memory suppression between age 8 and 12, through adulthood (Paz-Alonso et al., 2009). In addition, irrespective of age, a tighter coupling within a prefrontal-cingulate-parietal-hippocampal network was associated with more effective suppression (Paz-Alonso et al., 2013; for a related finding, see Yang et al., 2021). Memory suppression ability declines in older adults (Anderson et al., 2011), although this can be reversed by providing subjects with an appropriate strategy (i.e., direct suppression instructions; Murray et al., 2015). Hence, research in different cohorts has shown that cognitive control changes through lifespan according to an inverted “U-shape” curve. Besides age-related effects, it has been hypothesized that individual differences in regulating intrusive memories arise in part from pre-existing differences in executive function (cf. “executive deficit hypothesis”; Levy & Anderson, 2008), with important clinical implications.

Other studies have focused on psychological traits. For instance, higher trait anxiety was found to predict less successful suppression (Waldhauser et al., 2011). Another study found that individuals with high trait anxiety showed impaired memory suppression, especially for emotionally negative material, suggesting impaired cognitive control over aversive content (Marzi et al., 2014). In a study on self-perceived thought control abilities, participants with higher levels of perceived control showed greater forgetting than those with lower levels of control (Küpper et al., 2014). In addition, rumination has been associated with impaired SIF (Fawcett et al., 2015). Finally, higher retrieval suppression ability on a simple verbal TNT task predicted fewer distressing intrusions in the week after watching a traumatic film (Streb et al., 2016), and reduced impact of events scale scores, a measure of trauma. Hence, in healthy adults, psychopathological traits are associated with impaired SIF, whereas higher levels of perceived control are associated with increased SIF.

#### Clinical populations

Given its potential for investigating the relationship between cognitive control and psychopathology, the TNT task has been extensively adopted in a number of studies on clinical conditions. These include schizophrenia (Salamé & Danion, 2007), depression and dysphoria (Noreen & Ridout, 2016; Zhang et al., 2016; Sacchet et al., 2017; Noreen et al., 2020; Yang et al., 2020), borderline personality disorder (Sala et al., 2009), attention deficit hyperactivity disorder (ADHD; Depue et al., 2010), trauma and post-traumatic stress disorder (PTSD; Catarino et al., 2015; Waldhauser et al., 2018; Sullivan et al., 2019; Mary et al., 2020). Overall, these studies are consistent in showing impaired memory suppression ability in clinical populations, either in general or selectively for negatively valenced material (see Stramaccia et al., 2021, and Pevie et al., 2023, for quantitative meta-analyses of clinical studies using the directed forgetting procedure). These findings suggest that the TNT task may be a very useful instrument for clinical research, and a promising tool for treatment (e.g., Mamat & Anderson, 2023), especially if standardized procedures are adopted. Further evidence comes from non-clinical populations, whereby a greater history of trauma is associated with higher SIF for both negative and neutral memories than for those who report experiencing little to no trauma (Hulbert & Anderson, 2018). On the whole, given the amount of research that the TNT task has stimulated in the last twenty years, and its role in the characterization of memory control in different populations, this paradigm should be considered a flexible, generative tool with great potential in the field of cognitive control.

#### Emotional processing

The TNT task has been used to examine people’s ability to suppress emotional content. Direct suppression can inhibit negatively valenced memories, to the same degree as – or even more than – neutral material (Depue et al., 2006; Lambert et al., 2010; van Schie et al., 2013; although see Chen et al., 2012). Suppressing retrieval of unpleasant material triggers inhibition of mnemonic and emotional content (Legrand et al., 2021; Harrington et al., 2021; Nishiyama & Saito, 2022) and downregulates both the hippocampus and the amygdala in parallel, as established by effective connectivity^3^ (Gagnepain et al., 2017; for related findings, see Depue et al., 2007; Liu et al., 2016). Moreover, the negative coupling between the right DLPFC and these two structures was greater when unwanted memories intruded into awareness and needed to be purged (Gagnepain et al., 2017). Indeed, it has been argued that memory control may constitute a fundamental mechanism in cognitive emotional regulation, with relevant implications for psychiatric disorders (Engen & Anderson, 2018).

#### Autobiographical memories

Autobiographical versions of the TNT task (ATNT) have been devised (Noreen & Macleod, 2013; Stephens et al., 2013; Lu et al., 2023). In such a paradigm, participants are asked to generate autobiographical memories (possibly with differing emotional valences, if needed). Memories are then associated with personalized cues and learned to criterion. In the critical phase of the experiment, participants are shown the cues and asked to either recall or suppress the associated memory. By using this procedure, the authors observed evidence of suppression effects (i.e., greater forgetting of No-Think memories with respect to Baseline items; Section Critical pairs and fillers), either in terms of reduced specificity (i.e., poorer recall for details) or access to central details (but see Lu et al., 2023). Interestingly, the forgetting effect on both positive and negative autobiographical material observed immediately was not detected at follow-ups (both a few months and 1 year later; Noreen & Macleod, 2014; see also Mamat & Anderson, 2023); importantly, however, neither improved recall arising from retrieval practice on Think items, suggesting that in this paradigm at least, very large delays eliminate all single-session-induced modulations of memory recall, whether enhancement or suppression.

#### Episodic Future Thinking

The TNT task has also been adapted to study people’s ability to suppress fears about the future rather than memories of the past in a task known as the Imagine/No-Imagine procedure (Benoit et al., 2016; Ashton et al., 2020; Mamat & Anderson, 2023; see also Ryckman et al., 2018). Results showed that suppressing imagination engaged the right DLPFC, while inhibiting activity in the hippocampus and in the ventromedial prefrontal cortex. Notably, stronger inhibition in the latter region was associated with greater forgetting of details about dreaded events. In addition, suppression reduced feelings of apprehensiveness about feared scenarios. The Imagine/No-Imagine task is paving the way for investigating the clinical benefits of suppression training (Mamat & Anderson, 2023).

#### Anticipatory processing

The TNT task has been used to determine the effects of predictive precues on suppression-induced forgetting. Specifically, participants received (on a trial-by-trial basis) information about whether they were about to retrieve or suppress the memory associated with the upcoming cue (Section Precuing). Behaviorally, this approach showed stronger forgetting of suppressed trials when the precue was informative about the upcoming task, compared to when it was uninformative (Hanslmayr et al., 2010). Electrophysiological studies showed that this anticipatory process is accompanied by a decreased right frontal positivity, which predicted later forgetting (Hanslmayr et al., 2009), and by increased power in the theta frequency band in the DLPFC and higher long-range alpha phase synchronization (Waldhauser et al., 2015).

### Replicability of the Suppression-Induced Forgetting effect

In the last decade, “replication crisis” has been a hot topic in behavioral sciences, like in many other fields. Therefore, similarly to many other paradigms, the replicability of the SIF effect has also been discussed. To date, more than a hundred TNT studies have been published, the vast majority of which replicated the SIF effect (although with variable effect sizes), with one sizeable study representing 500 participants alone (e.g., Liu et al., 2021; Yang et al., 2021). However, there have been about a dozen failed replications (e.g., Bulevich et al., 2006; Meier et al., 2011), leading some to question the replicability of the SIF effect. Several critical factors while running a TNT study (e.g., quality of instructions, participants’ non-compliance, fatigue phenomena, etc.), as well as population differences (ageing, inhibitory control ability, psychopathological states, etc.) have been offered as potential accounts for these cases (Anderson & Huddelston, 2012). Notably, some personality traits (such as high trait anxiety and rumination) substantially worsen the SIF effect (Dieler et al., 2014; Fawcett et al., 2015). According to some, variability in the SIF effect may arise because true intentional forgetting necessarily requires an initial retrieval first that creates an urge to suppress, otherwise it will not take place (Wang et al., 2015; Section Waiting for the urge to suppress). A meta-analysis across 96 effects from 25 studies (Stramaccia et al., 2021) reported a large effect size for SIF (Cohen’s *d* = 0.66) in healthy participants who received direct retrieval suppression instructions, whereas such effects are substantially smaller in individuals who are affected by psychological disorders or who exhibit high scores on related traits (Cohen’s *d* = 0.17). Of note, a recent registered replication study using an online implementation of the TNT paradigm did not find a SIF effect (Cohen’s *d* ≤ 0.06), although the authors speculate that the online modality may have played a role in this outcome (Wiechert et al., 2023). Indeed, these same authors subsequently robustly replicated the SIF effect in participants run on the identical procedure, but in the laboratory (Wessel et al., 2023). Although this suggests that running the procedure online yields poorer quality data, successful online replications have been conducted (e.g., Mamat & Anderson, 2023), making the reasons for the lack of the SIF effect in Wiechert et al. unclear. Importantly, a multisite, multi-linguistic registered replication study on about 4000 participants from 21 countries, led by a committee of both key researchers and skeptics of the paradigm, is underway with the aim to formally assess the SIF effect (cf. Memory Control Consortium; Fawcett et al., 2023). We hope that this effort will add perspective to the inconsistencies in the field. Notably, although we provided informal consultation on this registered replication in its early stages (and also supplied a training video), we did not review the final protocol prior to its implementation. As such, we cannot comment on whether the procedure used in this replication adheres to best practices, based on our lab’s experiences.

Independent of the foregoing effort, we believe that providing a detailed, unambiguous, and thorough description of an experimental procedure plays a key role in any reliable replication. Far too often, pressure on journal space limits the description of the methods in psychological papers, so that they lack essential information to allow a reliable replication. In many cognitive tasks, simple things such as the phrasing of experimental instructions may have significant impacts on the outcomes. Therefore, our systematic report of the TNT experimental procedure will facilitate its standardization, reliability, and replicability.

## TNT task structure

Although the TNT task has evolved over the years, in that some aspects have been improved as a consequence of the scientific process, its core structure remains the same as in the original report (Anderson & Green, 2001). The TNT task includes three phases: learning phase, TNT phase, and recall phase. In the MATLAB functions provided with the present guide (cf. TNT training package), we label these phases “before training”, “training”, and “after training” to avoid inducing expectations of a later memory test in participants. Each phase is in turn structured into sub-phases (Fig. 1). The whole experiment takes about 1 h for the behavioral version, and 1.5 h for the fMRI version (40 min of which outside the scanner) per participant, excluding the administration of the post-experimental questionnaire (Section Post-experimental questionnaire (PEQ)) and debriefing (Section Debriefing).

### Learning phase

#### Study phase

In the study phase, we present the stimulus material to participants for the first time. Participants learn a list of paired items, often word pairs (but materials may change across different versions, and include material such as pictures; Section Words, Pictures). The pairs appear one at a time in the center of the screen with a “Hint” (or “Cue”) word on the left (or on the top) and a “Response” (or “Target”) word on the right (or on the bottom). Importantly, we tell participants that learning this material will be needed later for the core attention test. We carefully avoid any mention of a final memory test until the later recall phase (Section No mention of memory). We instruct participants to associate the Hint and Response members of each pair, so that they can recall the Response, given the Hint. We emphasize that they should study the pair for the entire amount of time it is displayed (Section Study phase), because they will be tested immediately after presenting the study list.

#### Test-feedback phase

After studying the pairs, participants proceed to the test-feedback phase. We tell participants that they will be tested to see how well they learned the pairs before moving on. Here, we present each Hint word in isolation, centrally, and instruct participants to name quickly and aloud the associated Response word of each pair (a microphone can be used to record responses). After a Hint disappears, we present the associated Response as feedback (typically in a different color; e.g., cyan) to reinforce knowledge of the pair, and we invite participants to take advantage of such feedback. We typically repeat this procedure up to two times (unless designed otherwise for specific reasons), or less if criterion is reached earlier. We normally exclude from the experiment those participants who are unable to reach learning criterion within two cycles. For populations with special needs (e.g., children, elderly, psychiatric patients), more cycles may be allowed. During this phase, the experimenter needs to score participants’ performance, either on paper or using a key press (Section Test coder). We score missing responses as “incorrect”. Also, we normally accept as “correct” responses given in plural form for singular targets (e.g., “dolls” for “doll”), and vice-versa.

#### Criterion test

The criterion test is the same as the test-feedback phase, but without the feedback. Here, participants try to recall all the pairs one last time. Again, the experimenter scores participants’ performance along the way, and we use such scores later for conditionalizing data analysis based on successful initial learning (Section Conditionalization). The same considerations for missing responses and correctness of singular/plural responses expressed above apply here.

#### TNT practice

The learning phase ends with practicing the TNT task. Practice gives participants a sense of the procedure and provides an opportunity for them to ask questions before the TNT phase begins. We administer the practice task twice and after each repetition we administer a diagnostic questionnaire (Section Diagnostic questionnaire (DQ)). We do this to verify that participants understand and are complying with the instructions received, and to correct them in case they do not (misunderstandings and non-compliance occur frequently at first). If the version of the TNT task adopted includes intrusion ratings (Section Intrusion ratings (practice)), we introduce this element during the second TNT practice cycle, after participants have built some familiarity with the basic task without intrusions ratings. Before the TNT practice, participants receive careful instructions about what they need to do during the TNT phase (Section TNT phase). Importantly, the TNT practice uses filler items and not critical items (Section Critical pairs and fillers). It has been our experience that including this TNT practice phase, with effectively delivered feedback in response to the diagnostic questionnaire, greatly improves data quality (see Dr. Anderson’s video for elaboration).

### TNT phase

#### Pair refresher

We normally skip this section, and it only occurs when considerable time (e.g., at least 15–20 min) elapses between the learning and TNT phases. This is often true in neuroimaging studies (e.g., fMRI, MEG, etc.) when participants need to be moved from one environment (testing room) to another (scanner), and general preliminary procedures unrelated to the TNT task occur (e.g., de-metaling, acquisition of structural MR scans, etc.). In such cases, we quickly show participants all pairs again (typically at a fast pace; Section Pair refresher) to refresh their knowledge of the pairs before moving to the TNT phase. Please note that, given that this section is optional, to run it one needs to enable the appropriate option in the main MATLAB function before starting the experiment (Section Running functions).

#### TNT phase

We tell participants that this section constitutes the core attention test. We tell them that they will be viewing Hint words, one at a time, either appearing in green or red (for pictures, green/red colored frames are normally used). We tell participants that when a Hint appears in green, their task is to look at it, think about the associated Response as quickly as possible, and keep it in mind the entire time the Hint is on the screen. When a Hint appears in red, instead, their task is to avoid thinking about the associated Response. We give further, detailed instructions for red trials, to make sure that participants understand and comply with the task requirements. Specifically, we ask participants to look at, comprehend, and attend to the Hint on the screen (i.e., pay full attention to it), until it disappears from the screen. At the same time, we emphasize that participants need to prevent the associated Response from coming to mind at all, not even for a second, and not even after the Hint disappears from the screen. We note that if the Response happens to come to mind, participants need to actively push it out of their mind. Critically, however, they should not replace the response with anything else (e.g., another word, image, or idea), a behavior that entails a mechanism of thought substitution rather than direct retrieval suppression (Benoit & Anderson, 2012; Section Direct suppression vs. thought substitution). Finally, we tell participants that the color in which each Hint is presented remains the same throughout the entire phase (i.e., Hints do not switch their color). We then warmly invite them to approach the task as a challenge and to do their best. It is also essential that participants are instructed to process the reminder “holistically”, that is, as a whole. To facilitate this, one needs to ensure that cues appear neither too small nor too large on the screen (Sections Words, Pictures). The TNT phase is typically composed of blocks (e.g., 4 or 5; Section Number of TNT blocks), interspersed with breaks of about 45–60 s to allow participants to relax and rest their minds, helping them to maintain their attention and focus during the whole TNT phase. Critically, within each block, each Hint appears several times (Section Number of TNT repetitions). The TNT phase (i.e., not considering TNT practice) typically lasts about 25 min for the behavioral version, and about 40–45 min for the fMRI version (Section TNT phase length).

### Recall phase

#### Study-context reinstatement and recall practice

The first step after the TNT phase is the study-context reinstatement task. Context reinstatement is adopted to reset participants’ mind and invite them to “mentally go back to the context in which stimuli were first learned” (Bäuml & Samenieh, 2010). Here, we prepare participants for the third phase, which will consist of recalling the associated Responses for all pairs learned in the learning phase. Because participants will have performed the TNT task for quite a while at this point, Baseline stimuli have not been seen for a long time at this stage (i.e., since the learning phase). We start by testing them on some filler items (Section Critical pairs and fillers) that were only used in the learning phase, but not in the TNT practice/phase. This helps participants to reinstate the study-context prior to the true final test (we actually use a mixture of Baseline, Think, and No-Think fillers in this task). At this stage, we first test participants with the original Hints; and then we test them with a new procedure, namely independent-probe testing (Section Independent-probe (IP) test). These introductory tests use filler items, whereas the true final tests that follow (same-probe and independent-probe testing) test critical pairs (Section Critical pairs and fillers). Participants are not told that these initial “introductory” tests are any different than the main tests, even though we treat them differently as experimenters (we don’t score them). As in the test-feedback phase and criterion test, the experimenter scores participants’ responses during the whole recall phase, either using a coding sheet or online using key presses (Section Test coder).

#### Same-probe (SP) test

In the same-probe testing procedure, we ask participants to recall the Response member of every critical pair learned in the learning phase, when provided with just the Hint member. We test all critical pairs, irrespective of whether they appeared in the TNT phase or not, and irrespective of whether their Hints had appeared in green or red during the TNT phase. Participants need to name the Responses aloud, and the experimenter scores these responses.

#### Independent-probe (IP) test

The independent-probe test has been an integral part of the TNT procedure since its inception (Anderson & Green, 2001). It allows the researcher to test for cue-independence, and to probe the presence of inhibitory processes and exclude associative interference and unlearning processes as the sole account of effects (Anderson & Spellman, 1995; Anderson & Neely, 1996; Section An active mechanism of forgetting). The rationale behind it is that if inhibition impairs the unwanted memory itself, recall should be worse regardless of whether an item is tested with the same cue used to train suppression (SP), or with a novel cue (IP). Therefore, in the IP procedure participants’ memory is tested with material that has not been used during the TNT phase. This can be done in different ways. A typical approach involves using an intrinsic semantic association between a (novel) Hint and a given Response (e.g., using “FOLDER–F” as cues to retrieve the target word “FILE”). An alternative approach uses a different episodic cue (independent of the original Hint-Response pairing) associated with the Response in the learning phase, but not used during the TNT phase. For instance, having learned an association between two Hints and the same Response word, TYPE–FILE and REST–FILE, one can use “TYPE” during the TNT phase and “REST” to independently test the Response word in the recall phase. IP testing is particularly relevant as it allows to test for cue-independence (Del Prete et al., 2015; Wang et al., 2015; but see Tomlinson et al., 2009 and van Schie et al., 2023 for discussions of this view), supporting an inhibitory interpretation of memory control processes, rather than associative interference (Section An active mechanism of forgetting). The order of SP and IP testing is normally counterbalanced across subjects.

#### Typical results

The typical results obtained in a TNT study are portrayed in Fig. 2. Memory performance at the recall phase is considered separately for SP and IP tests, as well as for Baseline, Think, and No-Think items (Section Critical pairs and fillers, and see Section Experimental conditions for a full explanation of their role in the TNT task). Based on these values, SIF (Section Suppression-Induced Forgetting (SIF), Suppression ANOVA) and facilitatory effect (Section Facilitation ANOVA) scores can be computed (Fig. 2A). When intrusion ratings are included in the protocol (Section Intrusion ratings (theory), Intrusion ratings (practice)), they typically show a declining trend as a function of the number of repetitions (i.e., suppressions) during the TNT phase (Fig. 2B).

### Notable changes to the standard procedure

Over the years, some strategic changes to the standard procedure have been introduced for special needs. Here are the most notable examples.

#### Study-list chunking

List chunking during the learning phase (e.g., dividing the material to be learned into shorter lists) should be considered when testing certain participants (e.g., children, depressed patients, older adults), who may have difficulty memorizing longer lists (e.g., Murray et al., 2015; Sacchet et al., 2017). When dividing the list into chunks, it is important to make sure that there is an even representation of the items that will be Think, No-Think, and Baseline items in each chunk. After all chunks have been learned to criterion, we generally present an integrated test of all items across chunks (see above references) to reduce differences in recency across sets.

#### Drop-off learning

Drop-off learning is a procedure in which test-feedback is limited to critical pairs that have not been learned. This implies that, as a participant goes through the test-feedback cycles, any Hint for which the associated Response is successfully retrieved is removed from the list, and only Hints that need repetition appear again. This procedure is mainly convenient when a high learning criterion (i.e., > 75–80%) is required, such as when the measurement of intrusions during the TNT phase is the major focus (e.g., Gagnepain et al., 2017; Levy & Anderson, 2012).

#### Precuing

A few studies have used instructional precuing to investigate anticipatory effects, that is, what happens when participants are pre-informed about whether the next cue will require retrieval or suppression (Hanslmayr et al., 2009, 2010; Waldhauser et al., 2015; Section Anticipatory processing). Typically, an instructional precue may be as brief as 1 s. Pre-cueing prepares a participant to retrieve/suppress in advance of seeing the cue. In doing so, it typically prompts a different mechanism than the one elicited in standard TNT protocols (i.e., proactive rather than reactive control; cf. Braver, 2012). This approach constitutes a major change to the standard procedure, and evidence suggests a strong impact on results (cf. Hanslmayr et al., 2009, 2010; Waldhauser et al., 2015), showing stronger forgetting for pre-cued No-Think trials, but also reduced forgetting for non-pre-cued ones. Because few studies have used this procedure and because of the latter unexpected finding, we recommend caution when implementing precuing, only using it when strictly needed in the experimental design.

#### Double-cue paradigm

In this procedure, two cues are associated with the same target during the learning phase. This approach has been used to disentangle inhibition and interference accounts of suppression-induced forgetting (Section An active mechanism of forgetting), showing that interference manipulations caused cue-specific memory impairment only for trained Hint-Response association, whereas direct retrieval suppression caused forgetting that generalized to the independent Hint-Response association (Wang et al., 2015).

#### Waiting for the urge to suppress

In this procedure, participants receive a substantially different set of instructions for the TNT phase. Namely, instead of starting to suppress right from the appearance of the Hint during No-Think trials, they are asked to wait until they sense the unwanted memory beginning to emerge – i.e., they wait until they have the urge to suppress to satisfy task goals. This procedure was introduced because according to some authors the emergence of an active memory that must be stopped is a required condition to engage an inhibitory mechanism (Wang et al., 2015; Section Measures of intrusion). According to this view, the key feature of a true inhibitory process is not “not thinking” in itself, but rather the suppression of the urge to retrieve a target memory. This mechanism would also explain failures in replicating the SIF effect (Section Replicability of the Suppression-Induced Forgetting effect), under the assumption that genuine intentional forgetting can only occur in trials whereby suppression is started immediately after an urge to retrieve a given memory, otherwise it would not take place.

## Options and parameters

### Stimuli

Several types of stimulus material have been used in TNT studies. Many studies have used verbal material (i.e., word pairs), but more and more studies have adopted visual materials including object-scene, face-scene, or word-object associations. Some studies have used personalized events, such as autobiographical memories or feared future events, associated to word cues.

#### Critical pairs and fillers

A typical stimulus set in a TNT study is made up of critical pairs and fillers. The former constitutes the core set of the study and are usually divided into three experimental conditions: Baseline (B), Think (T), and No-Think (NT). These are typically divided a priori into three matched sub-sets (e.g., three lists of word pairs: A, B, C; Section Words), which are assigned to experimental conditions in a counterbalanced manner across subjects (Section Counterbalancing pair sets). There are typically either 36 or 48 critical pairs (Section Number of critical pairs). Fillers are typically 18 and are used to control for primacy and recency effects, and to enable practice.

#### Words

Historically, word pairs were the first stimuli used in TNT studies (Anderson & Green, 2001; Anderson et al., 2004) and they remain widely used. We have created two standard sets of stimuli (one with 36 and one with 48 critical pairs) in English and made them available (cf. TNT training package). These sets have been created to ensure that semantic overlaps among the various items are minimized. This way, there is little inter-pair interference among Hints, Responses, and independent probes. Critical pairs are divided into three lists matched on basic features (e.g., average word frequency, word length, number of syllables, concreteness, relatedness, etc.). Standardized, translated materials, are also currently available upon request (and will be made publicly available in the near future) for 14 languages (Spanish, Portuguese, German, Dutch, Italian, French, Chinese, Japanese, Hebrew, Polish, Turkish, Russian, Swedish, and Hungarian) as a part of a multisite registered replication study (Fawcett et al., 2023; Section Replicability of the Suppression-Induced Forgetting effect).

Two variables that greatly affect item memorability are the concreteness of Hints and Responses, and the relatedness between a Hint and a Response. Indeed, high concreteness or relatedness can be purposely introduced to create customized sets to make learning easier (e.g., for children or clinical populations, to speed up the learning phase, or to increase the fraction of correctly remembered items at recall) or harder (e.g., to avoid ceiling effect). However, in general, most stimulus sets use pairs with weak or no relatedness, and moderate to low concreteness to avoid ceiling effects.

Another variable that needs to be considered when using words as stimuli is the font size when displayed onscreen. Indeed, the interplay between font size and viewing distance ultimately determines the visual angle covered by a cue. A key aspect of the TNT phase is that stimuli must be neither too small nor too large, but rather be processed holistically – i.e., as a whole. When stimuli are too small, participants may easily divert their gaze (during No-Think trials) to a slight eccentricity to avoid processing the Hint at all. When stimuli are too large, participants may instead attend to details (e.g., parts of the letters) and avoid perception of the whole stimulus, eliminating its tendency to cue the Response (see Dr. Anderson’s video for elaboration on this point). Thus, font size needs to be appropriate to ensure full attention to the whole word. Ideally, the stimulus should subtend a visual angle of 1 degree along the vertical axis. (Obviously, along the horizontal axis the stimulus subtends a much larger visual angle). The MATLAB functions we made available (cf. TNT training package) are optimized for a 20-inch monitor at about 60 cm from a participant’s eyes, and we chose a font size of 44-point. However, this parameter interacts with screen size and resolution, and the distance from which participants are watching. Hence, it should be measured on the specific system one intends to use, and if one plans to use substantially larger/smaller monitors and/or distances, one should consider adapting the font size accordingly. Issues of font size adaptation often arise in fMRI studies in which participants’ distance from the screen can vary widely from pilot studies outside the scanner to the experiment in the scanner. Given the potential of eye-tracking to monitor this aspect of participants’ compliance (i.e., overt attention), we recommend its adoption in future TNT studies (Section Eye-tracking).

#### Pictures

More recent studies adopting the TNT task have used pictorial material, mostly object-scene (e.g., Küpper et al., 2014; Catarino et al., 2015) or face-scene (e.g., Depue et al., 2006, 2007; Gagnepain et al., 2017; Sullivan et al., 2019) pairs. Typically, Hints are isolated objects or faces with a plain background, whereas Responses are naturalistic complex scenes depicting living or non-living objects in various environments. Such stimuli permit greater ecological validity and flexibility, allowing for the manipulation of emotional valence (e.g., negative vs. neutral; Gagnepain et al., 2017), or mimicking the cuing arising in trauma (e.g., traumatic situations; Catarino et al., 2015).

In the Recall Phase, participants are presented with Hints, and typically asked to provide a verbal description of Response scenes. They are normally given 15–30 s to do that, their descriptions are recorded, and then scored offline by the experimenter. The experimenter scores responses as correct if the scene has been described in a sufficiently detailed way to unambiguously identify the scene. Often, a second rater is employed to ensure high inter-rater agreement on coding decisions. The simplest scoring method is for the coder to render a binary “recalled” or “not recalled” decision based on whether they judge the description to be unambiguously referring to the correct scene (e.g., Depue et al., 2007). However, one can also score the level of detail provided and recall of gist (e.g., Catarino et al., 2015; Küpper et al., 2014).

Like the considerations made in the previous section, the size of cue picture display is also key. Again, stimuli should be neither too small nor too large. For pictorial material, based on our experience we recommend a visual angle about 3–4 deg.

### Timing

Timing is an essential aspect of the TNT task. The current standards reported below stem from our experience with the protocol over the years and are largely based on studies involving college-aged participants. Timing parameters may need to be adjusted when working with populations who substantially differ from college students, or for specific experimental requirements. Current recommended parameters are summarized in Table 1.

**Table 1:** Overview of current standard timing parameters in a TNT study. Numbers are expressed in seconds. Optional steps (and timings) are reported in brackets. ISI = inter-stimulus-interval

Parameter	Behavioral	fMRI
Learning criterion	50%	50%
Study cue	3	3
Study ISI	1	1
Feedback cue	4	4
Feedback response	2	2
Feedback ISI	0.5	0.5
Criterion cue	4	4
Criterion ISI	0.5	0.5
TNT practice cue	3	3
TNT practice ISI	0.5	1.4–2.6
[Pair refresher]	[1.5]	[1.5]
[Pair refresher ISI]	[0.8]	[0.8]
TNT cue	3	3
[Intrusion ratings]	[1.5]	[1.5]
TNT ISI	0.5	1.4–2.6
Retrieval cue	4	4
Retrieval ISI	0.5	0.5

#### Study phase

Our current standard timing for the study phase is 3 s per pair, with a 1-s inter-stimulus interval (ISI). Such durations constitute a trade-off among different needs: i) keeping the total length of the experiment within an acceptable time frame; ii) avoiding ceiling effects; iii) allowing enough time to reach a given learning criterion (typically 50%, but this may vary with a study’s specific needs and goals). In our experience, this timing accommodates most studies with college students as a target population. We have used exposure rates ranging from as low as 2 s (for high-performing populations), to as high as 6 s in populations with special needs. For instance, older adults or depressed populations might need longer learning times, but also longer ISIs, accompanied by solutions such study-list chunking (e.g., learning 20 pairs at a time before moving on; Section Study-list chunking).

#### Test-feedback phase

Our current standard for test-feedback timing parameters is 4 s for Hint presentation (i.e., seeing a Hint and naming aloud the associated Response), with a further 2 s for Response presentation (seeing the Response as a feedback), and 0.5 s for the ISI. Typically, the experimenter can press a key to skip any remaining bit of the Hint presentation (as well as the whole of the feedback) as soon as a participant names the associated Response. This is done to save time during the procedure and avoid getting participants bored or fatigued. Feedback duration can be reduced in high performing individuals to reduce ceiling effects.

#### Criterion test

Our current standard for criterion test timing is 4 s for Hint presentation, and 0.5 s for the ISI. Again, the experimenter can shorten a trial by pressing a button as soon as the correct Response has been provided, for the same reasons considered above.

#### TNT practice

Timing parameters for the TNT practice need to be the same as for the actual TNT phase (Section TNT phase).

#### Pair refresher

Whenever a refresher is part of the experimental procedure, the current standard for its timing is 1.5 s for Hint presentation, and 0.8 s for the ISI. The refresher is meant to be quick, hence longer timings should be avoided unless strictly necessary (e.g., when working with particular populations).

#### TNT phase

Our current standard for the TNT phase timing is 3 s for Hint presentation. Caution should be used when choosing longer durations, as sustained control may have consequences on intrusions, both during the trial at hand and during later ones, presumably due to a decline in control or fatigue (van Schie & Anderson, 2017). The ISI duration changes according to whether the study is a behavioral or an imaging one. In the former case, our current standard is a fixed ISI of 0.5 s to minimize the time available to experience intrusions after a stimulus disappears. Conversely, in fMRI a variable ISI ranging between 1.4 and 2.6 s (typically, with an exponential distribution) is recommended, to allow for jittering and therefore improving design efficiency (Henson, 2006). When intrusion ratings are included in the protocol, the maximum time allowed to respond is normally 1.5 s. If a response is provided before this time expires, the MATLAB functions automatically stop showing the intrusion ratings screen, and add any residual time (i.e., 1.5 – reaction time) to the ISI (i.e., fixation cross). Longer durations for intrusion ratings should be avoided (unless there are very good reasons to do so) to discourage participants from elaborately recalling the prior trial in making their judgments. Intrusion rating judgments should be quick, intuitive decisions about whether the item came to mind.

Importantly, an fMRI study will also include 20–33% of “null events”^4^, to improve design efficiency (Henson, 2006), which implies a longer duration (i.e., about 30% more time involving passive rest). The sequence and timing of events (including null events) used in the fMRI version of the MATLAB functions we provide have been optimized with OptSeq2 (https://surfer.nmr.mgh.harvard.edu/optseq/) using the following parameters: TR = 2 s., number of volumes = 200 (for 48 critical pairs) or 225 (for 36 critical pairs), FIR = 16 s., null events = 20%, and the contrast of interest (NT>T). If different parameters are to be used in a new study, a new trial ordering with OptSeq2 will be required. Choosing between behavioral and fMRI modes is easily made when launching our MATLAB functions by specifying either “0” (behavioral) or “1” (fMRI) as the input to the main TNT function in the MATLAB command window (see video #3).

#### Recall phase

Our current standard timing for Hint presentation during the test is 4 s, with ISIs of 0.5 s. Shorter durations have been used in some studies (e.g., 3 s) and longer versions.

### Intrusion ratings (practice)

We introduced the intrusion report to the TNT task later on (Levy & Anderson, 2012). In this procedure, during the TNT phase, we ask participants on a trial-by-trial basis whether they experienced the Response member for a pair coming to mind. Typically, we ask participants to press a different key to distinguish whether during the immediately preceding trial they experienced awareness of the memory: i) never; ii) briefly; or iii) often, and this is done on both No-Think and Think trials. Typically, options (ii) and (iii) are then collapsed to obtain a dichotomous response that can be used to compute a percentage (e.g., percentage of trials in which an intrusion happened on the first repetition, second repetition, etc.). However, we use a three-point scale only to encourage participants to report even brief intrusions. During intrusion ratings we strongly encourage participants to make their judgment quickly and intuitively, without consciously remembering the Response itself (Section TNT phase). We thus provide only a short time window to discourage participants from having time to think about/reinstate the associated Response, which may improve its memory. Participants experience awareness of the Response item during most Think trials, and show progressively declining intrusions (i.e., typically along an exponential distribution; Fig. 2B) during No-Think trials, as a function of the number of repetitions (i.e., suppressions; Section Number of TNT repetitions). Intrusion ratings capture an important aspect of memory control (Section Suppression-Induced Forgetting (SIF)), and their frequency and the steepness of their decline constitute important behavioral measures. Whereas SIF indexes a mnemonic aftereffect of suppression, intrusions provide an online index of the success with which people regulate awareness by suppressing the automatic retrieval of a memory. Although intrusion control is often correlated with the magnitude of SIF observed, these indices are sensitive to distinct processes.

## Instruments

In a TNT task the experimenter uses several standardized instruments to aid in running the study. Here we discuss the main ones, whereas others are introduced in the TNT training package (cf. TNT training package).

### Experimenter script

The main instrument is the experimenter script, which represents the backbone of the experiment. This script standardizes the administration of the whole experiment and contains instructions for both the experimenter and the participants. The instructions for the experimenter include reminders of the various steps and checks that need to be made, how to launch MATLAB functions, when to deploy other instruments (e.g., questionnaires), when to hand participants written instructions and when to score responses. These procedural reminders for the experimenter are read by them, silently, and only the experimenter must know them. The experimenter reads the instructions for participants aloud with participants following along on separate sheets of paper (Section Subject instructions). Elements written in shaded text in the experimenter script are instructions that should be read aloud to participants. Any text that is shaded and framed in black is meant to be read aloud to the participants and given to them on paper (for them to read). Text sections in all capital letters are instructions to the experimenter only, which are not meant to be read aloud. Here we provide a prototypical, standardized experimenter script (cf. TNT training package), which will be suitable for most cases. However, the script can be tweaked to accommodate specific requirements (i.e., different aims or experimental designs) when needed.

### Subject instructions

Subject instructions are made up of several sheets of paper, each of which is clearly referred to in the experimenter script. Each sheet contains a copy of the relevant instructions read by the experimenter and these sheets are meant to facilitate participants’ understanding. The experimenter hands participants each sheet of paper at a specific time and takes it back once those instructions have been given. This helps participants to focus on one thing at a time.

### Questionnaires

Two mandatory questionnaires are used in a TNT study.

#### Diagnostic questionnaire (DQ)

We verbally administer the diagnostic questionnaire (cf. TNT training package) at least twice during the TNT task, namely after the first and second TNT practice, at the end of the learning phase. A third (optional) administration may occur halfway through the TNT phase (e.g., between the second and the third TNT block). The goal of the diagnostic questionnaire is to make sure that participants understand and implement TNT instructions correctly, and to correct them in case they do not. It contains several questions, most of which focus on what participants are doing during No-Think trials (e.g., whether they look at Hint words, process them, try to suppress the associated Response, avoid thought substitution, etc.). The diagnostic questionnaire exists in two forms: one for the experimenter with a Likert scale, and one for the participant without it. Participants are asked for qualitative replies (so that they feel free to express themselves), and the experimenter translates their answers into numbers (together with taking notes if needed). Critically, the experimenter should take advantage of this opportunity to correct misinterpretations or incorrect attitudes on the participants’ side. The administration of feedback during the diagnostic questionnaire is a critical component of the TNT procedure, and we spend considerable time instructing our experimenters on giving effective feedback (Section Training researchers). Please, be aware that the administration of the diagnostic questionnaire is an important opportunity to reaffirm instructions, and praise (i.e., reinforce) participants for complying with instructions.

#### Post-experimental questionnaire (PEQ)

Another essential instrument for the experimenter is the post-experimental questionnaire (cf. TNT training package). We administer this questionnaire at the end of the experiment (i.e., after the recall phase), and it is devised to gather information about the way participants behaved during the study. This instrument can be adapted to flexibly fulfil various experimental needs (i.e., including further questions, see below). However, the core, mandatory part of the questionnaire measures whether participants ever made intentional efforts to think about Responses for Hints presented in red (i.e., No-Think trials; cf. question #2 of the post-experimental questionnaire). Three sub-questions probe participants about this aspect, and the individual scores of these need to be summed up to compute a collective score. If such collective score is 5 or higher, we exclude a participant from the study for non-compliance with instructions. Evidence shows that people who score 5 or higher exhibit a significant reduction (or even a reversal) in their SIF (Liu et al., 2021). Indeed, it is especially important to ask the first two questions, as a surprisingly high fraction of participants believe – despite the instructions received – that adopting such strategies is perfectly legitimate. We have found that if proper feedback is given during the diagnostic questionnaire phase (Section Diagnostic questionnaire (DQ)), very few participants will require exclusion based on this post-experimental questionnaire index of compliance (in a sample of 30, perhaps 1 or 2).

In addition to the diagnostic and post-experimental questionnaires, which are mandatory in any TNT study as they are linked to essential structural aspects of the task, a series of optional questionnaires may be included; e.g., enquiring about the strategies adopted during the TNT phase, demographic data, sleep habits (Section Hours of sleep) or alcohol and substance use. We normally collect such information (and strongly encourage other researchers to do so) and use it for data quality checks and to generate further hypotheses. In addition, other existing questionnaires of interest (e.g., trait anxiety, worries, rumination, perseverative cognition, meta-cognitive beliefs, mind-wandering, etc.) may be incorporated. However, the adoption of such questionnaires is study-specific and depends on the scientific aims at hand.

### Test coder

In the TNT task the experimenter needs to code participants’ responses during the test-feedback phase, criterion test, and recall phase. Normally, the stimulus sequence is pre-determined for each of these phases and kept constant across participants (Section Trial order). This allows the use of fixed lists with grids (cf. TNT training package) that can be printed and used to score responses. Based on our experience, we recommend a set of scoring practices: i) using a tick to score correct responses; ii) leaving a blank for missing responses; iii) writing down anything that deviates from correct responses. The most important thing is defining a protocol beforehand and sticking to it consistently across participants in a given study.

Computerized scoring is also possible using the MATLAB functions we provide (cf. TNT training package). This can be done by pressing online the “z” key for correct responses, and the “x” key for incorrect responses. The “space” key also can be used to truncate a trial as soon as a response has been provided (without coding “correct” or “incorrect”) and spare the residual time (skipping to the next trial). Using computerized scoring has pros and cons. It makes the calculation of the final scores easier, but it makes it harder to make corrections in case of accidental mistakes while coding. Our suggestion is to adopt computerized scoring only once the experimenter is experienced with the standard scoring procedure.

### Score sheet

At the end of the experiment, any scoring (whether on paper or computerized) needs to be translated into separate scores for Baseline, Think, and No-Think trials. This depends on the subject-specific counterbalancing condition assigned at the beginning. To facilitate this process, we provide a template score sheet (cf. TNT training package) wherein one simply enters ones for correct and zeros for incorrect responses and condition scores are calculated automatically.

## Designing experiments

We have identified several important screening criteria for the TNT task. One may not want to include them all in a study (especially because some of them may be factors of interest for those interested in individual differences). However, as a default we recommend including them.

### Screening criteria: demographics

#### Age range

Unless there is a specific hypothesis about development or aging, we recommend a default age range for participation as between 18 and 35. Elderly subjects (aged 65 and older) have a lessened ability to suppress memories (Anderson et al., 2011). Moreover, the specificity of the TNT instructions contributes to older adults’ suppression success (Murray et al., 2015; Section Individual differences). However, we have little data for intermediate age groups (i.e., middle-aged adults). Until data from intermediate age groups is available, focusing on a population unlikely to be influenced by aging effects is a conservative approach that could minimize variability in a given study.

#### Primary language

Our word stimuli are based upon norming studies designed for English native speakers, but stimulus sets in different languages exist (Section Words). To assure that our participants are uniform in the way they interpret the word pairs and perform the tasks, we require them to have spoken English as a primary language since early childhood (i.e., acquired before the age of 5). This decision is based on extensive experience. We have consistently observed that even people who are seemingly fluent in English experience difficulties in learning the word pairs if they have acquired English later than this, for instance resorting to encoding strategies that differ qualitatively from those of native speakers. This constraint is less relevant with pictorial materials.

#### Attentional abilities

People with attentional disorders (e.g., ADHD) are normally excluded, unless this aspect is specifically part of the experimental design. People with ADHD exhibit significant deficits in memory control (Depue et al., 2010). People with neurological conditions, brain injuries, and dyslexia are also excluded.

#### Color blindness

People with color blindness need to be excluded because the task relies on green-red color coding. A green-red color coding is typically used as it is a very intuitive and familiar reminder of traffic light color coding, which most people encounter in their daily life. Other color coding combinations (e.g., blue-yellow) may in principle be used, although they do not convey the same intuitive/familiar meaning. We are not aware of any published TNT study where a different color coding was used. Therefore, we recommend adopting the standard color coding (for uniformity with previous studies), unless there are good reasons to do otherwise.

### Screening criteria: performance

#### TNT-naïveté

Participants need to be TNT-naïve. TNT task instructions frame the experiment in terms of attention/resistance to distraction. This is because mentioning memory before the final recall phase may induce participants to disregard the core “No-Think” instructions given for the TNT phase, motivating them not to suppress during No-Think trials, and it has been shown that a lack of compliance with No-Think instructions significantly compromises the SIF effect (Liu et al., 2021). Once the experiment ends, participants know that their memory has been tested and, if they were to be recruited again for a similar study, they would likely not comply with No-Think instructions. Therefore, attention must be paid during recruitment to screen for students who might have already participated in previous studies. It is important to keep a list of people who have already participated in TNT studies in a given lab. Some college courses also include material on retrieval suppression that could render participants non-naïve.

#### Failing learning criterion

Another screening criterion is failing to reach the learning standard. The learning criterion (normally a minimum of 50% of the critical items for verbal material, but often higher for pictorial material; e.g., 90%) is set in advance. The logic is that if a participant does not learn the word pairs initially, there will be no need for them to suppress anything later. Also, the maximum number of test-feedback phases (typically two) needs to be set in advance. On this basis, participants who fail to reach learning criterion by the end of the second test-feedback phase are excluded from the study. However, many college students reach learning criteria within the first test-feedback phase, and most reach criterion within two, so only a very small minority is normally excluded on this basis (e.g., about 5%).

#### Non-compliance

Some people disregard the instructions to suppress during No-Think trials. We devised a questionnaire item that is essential in identifying non-compliant participants. We identify “cheaters”, that is, people who intentionally disregard instructions about No-Think trials with a core set of sub-questions (cf. question #2) in the post-experimental questionnaire (Section Post-experimental questionnaire (PEQ)). We exclude anyone scoring 5 or higher. However, the number of participants discarded based on this screening is low (i.e., less than 10%) if the proper precautions are taken to train experimenters and avoid references to memory during the experiment. Data from more than 500 participants show that the higher the cheating as indexed by this questionnaire, the lower (if not reversed) the SIF (Liu et al., 2021). This screening question is not intended to exclude participants who have difficulty in suppressing, but rather to find people who intentionally engage in behavior that violates the instructions. This distinction should be clarified to participants when they are answering this question. We also stress that at this point it is essential that they provide honest answers to questions, no matter what they have been doing during the TNT phase.

To lessen the number of non-compliant participants, we stress the importance of following the task instructions. This is why we administer the diagnostic questionnaire during the TNT practice (Section Diagnostic questionnaire (DQ)), which identifies situations in which participants are not following the instructions before the actual task onset. We have minimized cheating by carefully crafting our experimenter script and instructions to eliminate all mentions of “memory”, “memory tests”, and “studying” (Section No mention of memory). We also avoid memory-related terms in: advertising the study; labels on laboratory doors; consent forms; program names or file names; and stray books/materials around the testing room. Nothing evokes non-compliance like the idea that memory will be tested later. Indeed, the expectation of an impending test gives people a counter-motive to disobey the suppression instructions (i.e., to look “clever”; Section People must try to stop retrieval). This point is elaborated in the accompanying video by Dr. Anderson.

#### Hours of sleep

A final source of exclusion is the number of hours slept during the night preceding the experiment. We normally exclude anyone who did not sleep at least 5 h, as this may impact cognitive performance. Indeed, it has been demonstrated that sleep deprivation significantly reduces memory control capacity (Harrington et al., 2021). This issue is highly relevant in psychiatric populations, in which sleep is often compromised (Harrington & Cairney, 2021). To screen participants for sleep, we use a sleep questionnaire item included in the post-experimental questionnaire (cf. TNT training package; Section Post-experimental questionnaire (PEQ)).

### Circadian influence

Research suggests that inhibitory control ability may vary with circadian arousal, such that people tested during their “optimal period” are far better than those who are not (Ngo & Hasher, 2017). In two studies, we manipulated the time of the day during which people suppress memories (morning or afternoon) and found variation in inhibition (unpublished analyses on data reported in Anderson et al., 2011, and Murray et al., 2015). To avoid introducing circadian-related biases, we recommend to carefully control the time of the day in which participants are tested, when possible. For college students, the ideal time for testing is normally in the afternoon (e.g., from 12:00 pm to 6:00 pm), whereas for older adults it is in the morning. Critically, for between group comparisons, the time of day should be matched across groups, whatever time is chosen.

### Experimental design

In designing a TNT task, many design options can be chosen.

#### Experimental conditions

The number of experimental conditions in a TNT study is usually three: Baseline (B), Think (T), and No-Think (NT). All three item types are learned during the learning phase and recalled during the recall phase. During the TNT phase, T and NT items are repeatedly presented, and constitute the core experimental manipulation: T items get retrieval practice (and are generally better remembered at recall test), while NT items are suppressed (i.e., do not benefit from retrieval practice, and are generally worse remembered at recall test). Conversely, B items are omitted from the TNT phase and are key, as they constitute the reference against which both T and NT items will be compared on the recall test. Critically, B items are trained to the same degree of T and NT items during the learning phase. Further experimental conditions can be added. People have varied the number of suppressions (Anderson & Green, 2001), emotional valence of target items (e.g., Hulbert & Anderson, 2018; van Schie et al., 2013; Gagnepain et al., 2017), the trial duration of TNT trials (van Schie & Anderson, 2017) and many other variables. Adding conditions requires, however, that either the number of pairs to be learned is increased or the pairs per condition be decreased or both, which introduce other considerations that constrain the design, such as the prospects of participant fatigue, or variability due to small item sets.

#### Number of critical pairs

The number of critical pairs in a TNT study is a major methodological choice. Although one might think that the more, the better, a trade-off between statistical power and feasibility (i.e., the total duration of the TNT phase; Section TNT phase length) needs to be considered. Evidence shows that the minimum number of critical pairs per experimental condition is ideally at least 12 (i.e., 36 overall), otherwise variability may compromise statistical power (Jon Fawcett, personal communication). Fewer pairs can and have been used (as low as 5–6 per cell), but this usually necessitates larger sample sizes to compensate for the added error variability.

#### Number of TNT repetitions

Similar considerations apply to the number of repetitions of each item in the TNT phase. On the one hand, a larger number of repetitions is desirable to ensure a strong manipulation. On the other hand, the total duration of the TNT phase matters, due to fatigue (Section TNT phase length). Hence, a trade-off must be considered. This methodological choice is complementary to the previous one. A high number of critical pairs per condition will often need to be associated with a lower number of repetitions during the TNT task, and vice-versa (because increasing the number of pairs will increase during TNT phase duration). Earlier TNT studies investigated as many repetitions as 16 (Anderson & Green, 2001). Later studies reported that intrusion ratings decline and show a flattening after about the 6th-8th repetition, indicating that this index of memory control begins stabilizing at this point (e.g., Levy & Anderson, 2012). However, a minimum of eight repetitions seems needed to get reliable effects. Hence, we recommend using 12 repetitions with a set composed of 36 critical pairs, ten repetitions with a set of 48 critical pairs, and eight repetitions only when the number of critical pairs substantially exceeds 48.

#### Number of TNT blocks

Given a constant number of repetitions per TNT item (e.g., 12), one can organize the TNT phase into varying numbers of blocks (with an equal number of repetitions in each), interspersed by breaks (e.g., 1, 2, 3, 4, 6). The number of blocks chosen for the TNT phase is a trade off as well. Too many blocks would imply too many breaks, which might be distracting and inefficient. On the other hand, using very few blocks mean longer periods of time doing the task, this leads to greater tiredness/fatigue and not enough time to rest, which in turn would compromise performance. Our experience suggests that four or five blocks is the best option, depending on the number of repetitions chosen (e.g., 4 blocks work well with eight or 12 repetitions, whereas five blocks work better with ten repetitions). Breaks between blocks should last between 45 and 60 s, to allow participants to rest without losing focus. An optional diagnostic questionnaire may be administered halfway through the TNT phase (e.g., between the second and third block) to check that participants are still complying with instructions (Section Diagnostic questionnaire (DQ)).

#### TNT phase length

Caution should be used when exceeding a certain length for the TNT phase, namely about 45–50 min. Exceeding such length likely fatigues participants, leading to less careful compliance with task instructions and less control ability. This is especially true for vulnerable populations. Having said that, we have periodically used designs with college-aged students of up to 70 min (although it is preferable to stay below that threshold) when measurement or design issues demanded a longer TNT phase (e.g., Hellerstedt et al., 2016).

#### Trial order

Trials can be fully randomized or pseudo-randomized either in a subject-specific way, or in a pre-specified way for all participants. The latter approach simplifies some aspects of the procedure and has been adopted by many studies. The most effective approach is to pre-specify the specific order of items in the various sub-sections of the learning and recall phases (so that lists can be printed out for scoring, Section Test coder), and to create an “abstract structure” of the sequences used in the TNT phase (e.g., an example of an abstract sequence of conditions might be: T T NT T NT T NT NT T NT, etc.), which are then randomly filled-in with individual items in a subject-specific manner. This is how we sequenced items in the training material provided (cf. TNT training package). In behavioral studies, we also recommend ordering trials in the TNT phase so that there are no more than 2 or 3 trials of the same type in a row. This ensures that participants keep on switching between Think and No-Think trials, maintaining the need for active control. However, this guideline is somewhat different in fMRI studies (i.e., no more than four trials of the same type in a row), due to fMRI design efficiency requirements (Section TNT phase).

#### Counterbalancing pair sets

As already mentioned, (Section Critical pairs and fillers) critical pairs are typically divided into three fixed, matched sub-sets (e.g., sub-sets A, B, C), which are then assigned to experimental conditions in a counterbalanced manner across participants. This is done so that specific item-related and/or list-related biases are kept under control. Besides counterbalancing the assignment of sub-sets to experimental conditions, it is good practice to counterbalance across participants any other variable that might impact the results (e.g., the order of SP and IP testing in the recall phase; Section Independent-probe (IP) test).

Although item counterbalancing is the recommended practice for most TNT experiments, some laboratories may instead wish to randomly assign items to conditions for every individual subject. The rationale for doing so is that, given a high enough sample size, it is likely that every item will ultimately contribute to each condition equally, eliminating any biases across the Baseline, Think and No-Think conditions in the difficulty of the materials, just as item counterbalancing does. There are pros and cons to counterbalancing and randomization approaches. On the one hand, if one expects a very large sample size (e.g., > 100), the odds are good that both methods will match the representation of items across conditions. Randomization has the added advantage that a greater variety of item combinations occurs (e.g., that when item x is a Think item, item y can sometimes also be a Think item, but other times be a No-Think item) ensuring generalization of the phenomenon over many list configurations.

On the other hand, randomization is less ideal if: i) smaller sample sizes (e.g., 20–40) are being used; and ii) individual differences correlations are important to the study. With smaller sample sizes, the chances that randomization will accidentally introduce a bias in the difficulty of items contributing to different conditions grows to the point that counterbalancing is likely to be preferred. In the case of individual differences correlations (either with other behavioral or self-report measures, or with brain imaging data), counterbalancing is preferable. In most cases, researchers are interested in a subtractive measure such as SIF (Baseline – No-Think) that will be correlated with another measure of interest (e.g., stop signal reaction time, or activation in prefrontal cortex). However, this subtractive measure is not a pure index of the process of interest (e.g., inhibition), because it will also reflect differences in item memorability across conditions. For example, imagine that Baseline items are very easy, but No-Think items are very hard; in this case, one would observe a massive difference score, with Baseline recall performance much higher than No-Think recall, even if no inhibition whatsoever had occurred. Such a score would potentially obscure any predicted relationship between SIF and other measures of inhibitory control, as the score would be taken to indicate high inhibition when none had occurred. The chances of such extreme differences in item memorability between conditions are particularly high given the relatively small number of items contributing to each condition, a situation made necessary by the intrinsic difficulty in memorizing large numbers of word pairs. When item-counterbalancing is used, one can estimate the contribution of item memorability differences and statistically account for them, because many participants will share the precise item assignments that a given participant has (Section Z-normalized scores). With randomization, this correction is not as readily done, as no two subjects will have identical assignments. For these reasons, we strongly advise item counterbalancing when interest focuses on how individual variability relates to other behavioral or brain measures.

#### Sample size

Finally, one needs to determine the desired sample size for a given study. Based on the average effect size for SIF (Cohen’s *d* = 0.66) in healthy participants who received direct retrieval suppression instructions (Stramaccia et al., 2021; Section Suppression-Induced Forgetting (SIF)), we ran a power calculation with the following parameters: two-tailed test, within-subjects comparison, α = 0.05, power = 0.80. This returned a sample size of 21 participants. However, besides this, it is good practice to aim for a sample size that is an integer multiple of the number of counterbalancing conditions. For instance, if one has three lists (A, B, C), and two SP/IP orders (corresponding to 6 counterbalancing combinations), one might aim to round the number up and collect either *n* = 24 or *n* = 30 to allow perfect counterbalancing, or any multiple that most closely achieves the desired power.

## Running experiments

### Setup

#### Experimental environment

Care must be taken when preparing a room to run a TNT study. Besides general issues such as choosing a quiet place, where participants do not get distracted by interfering stimuli, the experimenter should also ensure that the environment does not contain mentions of “memory”. As we have discussed (Section TNT-naïveté), participants must be naïve with respect to memory testing to avoid non-compliance. Care must be taken to avoid environmental items that might accidentally induce expectations about a later test (Section Non-compliance). Examples include papers about memory on a desk, books about memory on the shelves, a poster presented at a conference on memory on the wall, or a plaque with “memory lab” on the room door.

#### Preparing material

During a TNT task the experimenter will need paper sheets at hand, including informed consent, experimenter script, subject instructions, diagnostic questionnaires, post-experimental questionnaire, test coders, debriefing sheet, etc. (cf. video #2).

### Instructions

#### No mention of memory

Related to Section Experimental environment, the experimenter must take care not to mention anything related to “memory” or “memory testing” before the final phase of the experiment (recall phase; Section Non-compliance). Instead, the instructions introduce the study as being about attention and resisting distraction. Several reminders of this essential point appear in the experimenter script (Section Experimenter script). Consistently, all advertisements released in advance should be phrased in these terms, without mentioning memory. This is done as it has been shown empirically that a lack of compliance with No-Think instructions significantly compromises the SIF effect (Liu et al., 2021). Hence, participants must be blind to the main experimental manipulation, as its awareness might encourage non-compliance.

#### Standardization of procedure

The TNT task procedure is complex, composed of several phases and sub-phases. For this reason, it is important that its execution is precisely defined, and that the experimenter follows a standard procedure. This is why the experimenter should use instruments such as the experimenter script and subject instructions (Sections Experimenter script, Subject instructions). Following the scripts ensures that nothing is overlooked, that exactly the same procedures and instructions are used with all participants.

### MATLAB functions

#### System requirements

The MATLAB functions provided (cf. TNT training package) have been written using MATLAB 2017a (www.mathworks.com) and PsychToolbox 3 (http://psychtoolbox.org). Although these functions will work on many systems, their functioning and stability need to be tested on the specific system one intends to use to run the study and modified if needed.

#### Running functions

We have created a tutorial video to show how to launch the MATLAB functions to run the TNT study (cf. video #3 in the TNT training package). The main function (called “tnt_standard_script”) needs to be launched three times, corresponding to the three main phases of the TNT task, i.e. “before training” (learning phase), “training” (TNT phase), and “after training” (recall phase). When launching the main function, one needs to specify whether the study needs be run in behavioral (“tnt_standard_script(0)”) or fMRI mode (“tnt_standard_script(1)”). At the beginning of the session, one needs to input a participant’s ID and the counterbalancing options (Section Counterbalancing pair sets). Within each main phase of the TNT task, the various sub-sections simply proceed by the user clicking the left mouse button. Methodological options such as including intrusion ratings or a pair refresher can be modified by changing a few parameters in the main function (cf. comments in the MATLAB code).

### Interacting with participants

In a TNT task, the experimenter needs to closely interact with participants on several occasions, and the experimenter’s presence in the testing room (focusing on participants’ activity) is a factor that boosts participants’ engagement.

#### Welcome and introduction

When welcoming participants, besides creating a positive rapport and making sure that they feel comfortable and at ease, one should minimize interruptions and distractions (e.g., mobile phone) for the whole length of the experiment.

Given the importance of compliance with task instructions, it is essential that participants view the task as important enough to sustain their effort for a prolonged time. One way to convey the importance of their participation is to frame any behavioral study as a pilot for fMRI studies, which are expensive and highly demanding. For these reasons, we include, in the introductory remarks to the script, a description of how important their participation is in helping to conduct pilot work for such an imaging study (experimenter script; Section Experimenter script).

#### Scoring responses

As we have mentioned (Section Test coder), there are times in a TNT study when the experimenter needs to score participants’ responses (during test-feedback phase, criterion test, and recall phase). Irrespective of whether such scoring is done using paper sheets or key presses, the experimenter should avoid distracting participants with movements/noise. Experimenters sit a bit behind and beside participants, so they are not distracted by scoring (cf. video #2 in the TNT training package).

#### Administering questionnaires

As mentioned (Section Diagnostic questionnaire (DQ)), the diagnostic questionnaire is administered in a verbal, interactive manner (i.e., read aloud by the experimenter, who also makes notes, while participants follow the questions on a sheet of paper). The focus is more on getting qualitative feedback from participants, rather than translating their behavior into numbers. We would like to stress once more the importance of the diagnostic questionnaire as an opportunity to reaffirm instructions and encourage participants to comply with them.

The post-experimental questionnaire should be administered by the experimenter interactively. The interactive nature is particularly important when administering the question about cheating, because the experimenter should make it clear to the participant that: i) we are concerned with intentional efforts to bring a memory to mind, and not any lapse in memory control; and ii) they should be as honest as possible, as this will help us.

#### Answering questions

Participants often ask questions about instructions. Examples of recurring questions can be found in video #2. Typical questions concern what is allowed during No-Think trials. While some instructions are given in this regard (e.g., attending and processing all Hints for their whole duration, not replacing associated Responses with any other image, thought or idea), the experimenter should refrain from suggesting specific strategies. This is also why it is both useful and important to debrief participants about their strategies, via the post-experimental questionnaire (Section Post-experimental questionnaire (PEQ)).

#### Monitoring compliance

Compliance with instructions can (and should) be monitored in several ways. One approach is using both the diagnostic (Section Diagnostic questionnaire (DQ)) and post-experimental questionnaires (Section Post-experimental questionnaire (PEQ)). Another is to adopt the seating suggested in Section Scoring responses during the whole TNT task, so that participants get the impression that their behavior is monitored. In addition, eye-tracking can be used, if available (Section Eye-tracking), to ensure that participants’ attention remain on cues during the TNT phase.

#### Managing breaks

The main breaks in a TNT task are those between TNT blocks during the TNT phase. Such breaks are meant to make participants relax and rest their minds. They range between 45 and 60 s. Participants need to stay focused on the TNT task, and long breaks would prolong the TNT phase beyond the recommended maximum time (Section TNT phase length). During such breaks, participants should be mostly left alone, rather than engaged in casual chatting. However, a few words of praise for the effort made, and encouragement are useful to keep participants motivated. It is also often useful to have a short comfort break between the end of the learning phase and the beginning of the TNT task.

### Debriefing

At debriefing, we tell participants about the aim of the TNT study, namely the investigation of their memory control ability (a template of debriefing is provided; cf. TNT training package). A debriefing also constitutes a good opportunity to gain insights into what participants were doing during the task. In the post-experimental questionnaire (Section Post-experimental questionnaire (PEQ)) we include optional questions to gather information about the specific strategies adopted in the TNT phase, and sometimes we also ask open questions. Over the years, this approach has made us aware of non-obvious aspects of the task and has helped us generating new scientific questions.

Debriefing is also the right time to discuss the deceptive aspect of the TNT task, namely the lack of any mention of memory testing before the recall phase. Some ethics boards, in fact, consider this as deceptive, and therefore this aspect should be fully disclosed here. However, it should be noted that such deception: i) is only partial, as we frame the task in terms of attention/resistance to distraction, which is also true (we only omit the bit about memory); ii) is absolutely necessary, as sharing such information with participants would compromise compliance and the SIF effect (Section No mention of memory); iii) poses only a minimal risk to the participants; iv) is fully disclosed at the end of the experiment.

## Practical guidelines

Because this topic is treated extensively in Dr. Anderson’s lecture (cf. video #5 in the TNT training package), we revisit these issues only briefly here. Three theoretical preconditions must be met to have a reasonable expectation of observing the SIF effect. Experimenters need to be aware that certain behaviors participants may engage in, as well as certain elements in the experimental design, can undermine (i.e., violate) such theoretical preconditions. In a manner similar to the stop-signal task (Logan & Cowan, 1984; Band et al., 2003), we can think about No-Think trials in a TNT task as prompting a “horse race” between a retrieval process (triggered by the specific content of a Hint) and a suppression process (triggered by the color of the Hint) that aims to prevent a memory from entering awareness. Given this framework, the three preconditions are as follows.

### Reminders must be attended

In a TNT task, it is essential that reminders (i.e., Hints) are attended during Think and No-Think trials. If participants do not attend to reminders, the retrieval process is not triggered. Hence, there would be no retrieval process to stop, no memory to inhibit, and consequently no SIF should be observed. Indeed, in everyday life it is natural to avoid looking at reminders of unwanted memories (e.g., getting rid of an ex-partner’s pictures). Participants can do that in a TNT task by slightly diverting their gaze to one side of a stimulus (or just above or below), or by focusing attention to the space in-between the letters, therefore avoiding processing the reminder at all, or redefining its nature. Several solutions counteract this problem. First, special attention should be given to stimulus size (i.e., its visual angle), making sure it is neither too small nor too big, but rather just appropriate to be processed “holistically” (i.e., as a whole; Section Words). Second, the presence of alternative salient foci in the environment (e.g., a reflection on the screen) should also be checked and fixed. Third, the need to keep one’s attention on the reminder needs to be emphasized when giving instructions, using feedback during the TNT practice/diagnostic questionnaire. Fourth, eye-tracking can be profitably used to monitor oculomotor behavior (Section Eye-tracking). Finally, we believe that careful training of researchers aimed at ensuring full attention to cues is key (Section Guidelines for best practice).

### People must try to stop retrieval

To observe suppression-induced forgetting, retrieval stopping must be attempted. In a TNT task there is no natural motivation for participants to stop retrieval because the to-be-suppressed content is often not unpleasant or of personal significance. The only motive participants have is social desirability, the need to feel like one is cooperating with another person’s requests. Indeed, participants often have counter-motives (i.e., motives to do the opposite of instructions), such as the desire to appear clever or to avoid deception if they understand that their memory will be tested later on (and this is especially applicable to college students). So, sometimes people not only fail to stop, but also rehearse the No-Think items, in violation of a fundamental precondition of the effect.

Other times, failure to engage in retrieval stopping might stem from a lack of understanding of the precise instructions, leading participants to believe that some mental acts are perfectly legitimate. For example, with surprising frequency, people believe that briefly checking that their memory of the response word is intact prior to suppressing the word is consistent with the instructions; others think that simply letting the word drift out of awareness if it intrudes is acceptable; and others think that checking their memory for No-Think items in between trials is acceptable.

Different countermeasures can be put in place for different problems. Countermeasures for the lack of motivation involve creating an atmosphere of respect, courtesy, and consideration (e.g., be on time, know participants’ names, make sure they are comfortable, make them understand that their help is very much appreciated), and creating an atmosphere of importance (be dressed in a certain way, giving importance to what is being done, etc.). Countermeasures for the presence of counter-motives can be the elimination of any memory framing and terms (e.g., study, test, retrieval, etc.), and reframing it as being about attentional ability and the ability to ignore distractions. Framing the task as being about the ability to ignore distraction aligns participants’ inner motivation to feel smart with the TNT task goals (Sections Experimental environment, No mention of memory). Finally, the main countermeasures to a lack of understanding of the instructions are to: i) eradicate wrong behaviors while giving instructions, that is, capitalizing on TNT practice and the diagnostic questionnaire (Section Diagnostic questionnaire (DQ)); and ii) screening for compliance using the post-experimental questionnaire (Section Post-experimental questionnaire (PEQ)).

### People must be able to inhibit

The capacity to inhibit must be present in participants. Many factors may violate this precondition, even if retrieval stopping is attempted. For instance, state-related variables such as boredom, fatigue, (e.g., exam-related) and stress can undermine memory control (Harrington et al., 2021; Quaedflieg et al., 2020, 2022; Ashton et al., 2020). Other variables can be ascribed to experimental design or procedural flaws: a lengthy TNT phase; a lack of short breaks; the order of TNT tasks among other tasks; uncontrolled sources of distraction (e.g., noise, heat, thirst, feeling uncomfortable, biological necessities, etc.). Countermeasures to these issues include: screening participants for sleep deprivation (Section Hours of sleep); avoiding morning sessions (especially with college students; Section Circadian influence); keeping the TNT phase within a reasonable time frame (Section TNT phase length); giving appropriate short breaks (Section Managing breaks); always running the TNT task first if a study includes many tasks; making sure that participants are comfortable and ready to start (Section Welcome and introduction).

## Data analysis

The main dependent variable in a TNT task is the percentage of correctly recalled items per experimental condition (B, T, NT). This is computed separately for same-probe (SP) and independent-probe (IP) testing (Sections Same-probe (SP) test, Independent-probe (IP) test; Fig. 2A). Besides this, measures of intrusion are also used (Section Intrusion ratings (theory), Intrusion ratings (practice), Measures of intrusion; Fig. 2B).

### Conditionalization

#### Unconditionalized data

In an unconditionalized dataset, the percentage of recalled items per experimental condition is calculated without considering the critical pairs that were learned. For instance, when adopting a stimulus set of 48 critical pairs, the total number of pairs per condition is 16. If a participant scores ten correct responses out of 16 total items (e.g., in the No-Think condition), the corresponding percentage will be 62.5%. This procedure has pros and cons. The pros are that the scores can be calculated exactly on the same stimuli across all participants, and that ceiling effects are less likely than in the conditionalized analysis (see below). The con is that the scores for No-Think items mix forgetting due to suppression with omissions due to lack of learning. If participants learn No-Think items particularly poorly, for example, the measure of SIF (Baseline – No-Think) will be inflated by the poorer learning in the No-Think condition; conversely, if the Baseline items are particularly poorly learned, the SIF measure will be artificially deflated underestimating true SIF.

#### Conditionalized data

In a conditionalized dataset, the percentage of recalled items per experimental condition is calculated considering only the critical pairs that were learned, as established at criterion test (Section Criterion test). Following the example above, if a participant scores ten correct responses out of 13 learned items, this time the corresponding percentage will be 76.9% (notice that the numerator is the same, whereas the denominator is smaller). This procedure also has pros and cons. The pro side is that estimates of SIF are less likely to be contaminated by differences in the amount learned across Baseline and No-Think items, contributing to a better estimate of this effect. Moreover, conditionalizing increases the robustness of fMRI activations in neuroimaging studies of retrieval-suppression (Anderson et al., 2004). For example, by eliminating unlearned items, fMRI activations of dorsolateral prefrontal cortex during No-Think trials increase because “effortless” No-Think trials are eliminated in which participants do not have to suppress anything; and “effortful” Think trials are eliminated in which people struggle to recall Think trials they didn’t learn. The cons are that: i) the scores are calculated on slightly different stimuli across participants; and ii) a ceiling effect is more likely, at least on immediate tests (i.e., compressing the estimation of the SIF, especially when using the SP test; Section Same-probe (SP) test).

### Suppression-Induced Forgetting (SIF)

The SIF effect refers to the typical outcome of a TNT experiment (Sections An active mechanism of forgetting, Typical results), whereby the percentage of correctly recalled items in the No-Think condition is significantly lower than the percentage of correctly recalled Baseline items (Fig. 2A). The magnitude of SIF may vary across studies, but it is typically about 7–10%. A recent meta-analysis across 25 studies reported a large effect size for SIF (Cohen’s *d* = 0.66) in healthy participants who received direct retrieval suppression instructions (Stramaccia et al., 2021). For the sake of simplicity (i.e., to interpret results in a more straightforward manner), when analyzing the data of a TNT study, we suggest running two separate ANOVAs focused on distinct a priori effects.

#### Suppression ANOVA

In a suppression ANOVA, the percentage of correctly recalled items is compared between No-Think and Baseline conditions. This comparison tests the SIF effect introduced above, and there is a strong prediction that a significant SIF will be found (i.e., B>NT), indicating an inhibitory effect of retrieval suppression. In such an ANOVA, counterbalancing is usually entered as a covariate (Section Covariates).

#### Facilitation ANOVA

In a facilitation ANOVA, the percentage of correctly recalled items is compared between Think and Baseline conditions. Here, there is a weaker prediction of observing a facilitatory effect in the recall of items in the Think condition, compared to the Baseline condition. In fact, some studies have reported a significant facilitatory effect (e.g., Anderson & Green, 2001; Anderson et al., 2011), whereas some others have not (e.g., Castiglione et al., 2019; Catarino et al., 2015; Levy & Anderson, 2012), and a few have even observed a numerical reversal (e.g., Benoit et al., 2016). Various interpretations have been provided to explain such phenomenon (e.g., Paz-Alonso et al., 2009). One is that, typically, Baseline and Think trials are both at ceiling, hence their relative difference (i.e., magnitude) may be reduced, sometimes approximating zero. In addition, facilitation effects for Think items can be especially small when the Response item in a pair is a complex stimulus, such as a scene, which may be very difficult to fully retrieve during Think trials (e.g., Catarino et al., 2015). If complex Think items are only partially retrieved, elements that remain unretrieved may be forgotten by retrieval-induced forgetting, which will negatively affect any quantitative measure of details recalled.

Another explanation, typically discussed in the context of IP testing, is that the lack of a facilitatory effect arises from the “encoding specificity principle”, whereby cues available at retrieval are more effective when they are similar to the conditions present at encoding (Thomson & Tulving, 1970; Tulving & Thomson, 1973; Murphy & Wallace, 1974). Thus, repeatedly retrieving the association between cue A (e.g., River) and target B (e.g., Bank) may increasingly bias the representation of target B towards a meaning consistent with A (e.g., the side of a river), making it harder to recall from independent cues that may not share that bias (e.g., “Money - B_”; see Paz-Alonso et al., 2009, for a discussion of this hypothesis).

Because the facilitation effect is usually not the central reason for conducting a TNT study, and because this effect has distinct mechanisms contributing to it, we advise separating this analysis from the one of central importance and discussing it separately.

#### Covariates

If counterbalancing is chosen instead of randomization (Section Counterbalancing pair sets), then counterbalancing conditions should be included as covariates in the above-mentioned ANOVAs to account for item- and/or list-related effects. Indeed, whenever interactions between these and SIF are suspected (i.e., most situations, which can add noise to the data), counterbalancing should be preferred to randomization, as the former can be included in the analysis as a factor, whereas the latter cannot. Including counterbalancing as a covariate allows researchers to account for differences in item memorability, resulting in greater accuracy of the statistical model (i.e., greater statistical power by reducing the error term; cf. Pollatsek & Well, 1995), and hence in greater reliability of the results (i.e., the ANOVA results will have been cleaned up of memorability effects, and their interpretation will be more straightforward). Counterbalancing conditions include the assignment of lists (A, B, C) to experimental conditions (Sections Critical pairs and fillers, Counterbalancing pair sets), and may include the order of SP/IP testing in the recall phase (Section Independent-probe (IP) test).

### Measures of intrusion

We operationally define an intrusion as an involuntary retrieval of a given memory despite an active effort to keep that memory out of awareness. Intrusion measurements were introduced in the TNT task to track participants’ success/failure at stopping unwanted memories from coming to mind on a trial-by-trial basis (Section Intrusion ratings (theory)). Often, intrusion measures show a clear relationship with SIF (e.g., Levy & Anderson, 2012; Hellerstedt et al., 2016). However, is not necessarily the case, as intrusions may reflect different aspects of memory control (Levy & Anderson, 2012; Anderson et al., 2016). For instance, memory retrieval could be stopped before any intrusion occurs (e.g., by a proactive mechanism; Section Precuing), and before inhibition of hippocampal activity occurs. Accordingly, whereas SIF would index inhibitory control, intrusions may represent a mixture of accumulating inhibition over time, and a mechanism of retrieval prevention (Levy & Anderson, 2012; Anderson et al., 2016; Crespo-García et al., 2022). In a TNT task, we typically use two measures of intrusions, intrusion frequency and intrusion slope.

#### Intrusion frequency

Intrusion frequency refers to the number of intrusions that participants experience during the TNT phase. Typically, they can be quantified overall (i.e., during the whole TNT phase), per TNT block, or per individual repetition (e.g., first suppression attempt, second suppression attempt, and so on, like in Fig. 2B). Evidence across many studies shows that intrusions undergo a systematic decline across suppression attempts (e.g., starting at a frequency of about 60%, and then showing a proportional reduction of nearly 50%; e.g., Levy & Anderson, 2012; Benoit et al., 2015; Gagnepain et al., 2017; Harrington et al., 2021; Hellerstedt et al., 2016; van Schie & Anderson, 2017).

#### Intrusion slope

Intrusion slope refers to the steepness of the curve characterizing the decline in intrusions as a function of the number of suppressions (Sections Intrusion ratings (practice), Number of TNT repetitions). This measure captures the efficiency with which memory control mechanisms purge intrusions with effort (Levy & Anderson, 2012). Unlike intrusion frequency, it characterizes the dynamic of intrusions (i.e., whether increasing or decreasing, and the speed of their reduction) and so captures important additional information. Indeed, a given overall intrusion frequency (e.g., 50%) can come about in different ways, including starting off with 100% intrusions and declining to 0%, or the reverse, two outcomes that would have very different interpretations. Intrusion slopes are usually proportionalized (Section Proportionalized intrusion slope).

### Data transformation

#### Z-normalized scores

It is important to z-normalize SIF scores within participants’ counterbalancing groups whenever correlations with individual differences need to be computed (e.g., Levy & Anderson, 2012; Hulbert & Anderson, 2018). This analysis step controls for differences in memorability and intrusiveness of items in each counterbalancing group (Sections Counterbalancing pair sets, Covariates), by quantifying how unusual a participant’s inhibitory ability is with respect to a homogenous group of participants receiving the same items in the same conditions. For example, imagine that item sub-sets 1 and 2 differ in their Baseline memorability – where the average Baseline recall of 1 is 70%, the average Baseline recall of 2 is 50%. Now further imagine that item sets 1 and 2 are assigned to the Baseline and No-Think conditions in counterbalancing group A, but the reverse in Counterbalancing group B. In counterbalancing Group A, there is a “built-in” 20% deficit in the No-Think condition compared to the Baseline condition, prior to participants even doing any suppression. This difference, however, is clearly due to item variability and not SIF. In contrast, the reverse is true in counterbalancing group B. To prevent such “item variability” from contributing in misleading ways to individual differences correlations, we first z-normalize all subjects’ SIF scores (B–NT) within a particular counterbalancing group (e.g., Group A). This z-score then expresses how unusual each participant’s SIF score is relative to all other subjects with those same items assigned to the same conditions. The same is done within each of the counterbalancing groups. The resulting z-normalized scores can then be entered into correlations with other measures, with less concern about variance due to item differences. Accounting for such item variability is essential whenever individual differences are the focus of the study.

#### Proportionalized intrusion slope

To compute a proportionalized intrusion slope, we first divide the number of intrusions at each time point (e.g., TNT block) by the intrusion rate from the first repetition (e.g., Levy & Anderson, 2012). A slope is then computed over these proportionalized scores. This proportionalization is done to account for the fact that initial intrusion rates can vary greatly across participants. This variation in the number of intrusions at the outset creates a problem because participants with more initial intrusions will have greater room to decrease their intrusion frequency over time than participants who are very successful in intrusion regulation from the outset. Without proportionalization the intrusion slope represents an absolute decrease in the level of intrusions; with proportionalization, the intrusion slope considers the amount by which intrusions could in principle drop (the start point) and then expresses the efficiency of intrusion control, relative to that starting point. Thus, someone who starts with 60% intrusions and reduces intrusions to 30% would have the same proportionalized intrusion slope as someone starting at 30% who then reduces intrusions to 15%. So, by proportionalizing, we correct for the potential for reduction and level it out across participants. Based on many datasets collected in our lab, we observed that proportionalized slopes often correlate with SIF and other measures of inhibition better than do raw slopes. Therefore, we recommend that researchers use proportionalized slopes when computing correlations.

## Summary and limitations

### Best practices

#### Guidelines for best practice

At this point, we would like to sum up what we believe are the most important guidelines for good practice when running a TNT study. In a nutshell:

1. Frame the study as being about the ability to pay attention and avoid distraction;

2. Avoid any reference to memory (testing) in any context;

3. Ensure adequate Hint-Response associations (i.e., reaching a pre-established learning criterion);

4. Use a two-iteration TNT practice with fillers;

5. Use diagnostic questionnaires after each practice;

6. Carefully check the TNT cue size;

7. Insert short breaks (45–60 s) during the TNT phase;

8. Use ten or more No-Think repetitions;

9. TNT phase should last ideally < 45–50 min;

10. Screen for non-compliance;

11. Avoid factors that would compromise attention to task;

12. Carefully train researchers.

#### Training researchers

We believe that standardized training is essential to administer the TNT procedure effectively. Our hope is that the tools we provide here (i.e., main text and TNT training package) will increase the standardization, reliability and replicability (Section Replicability of the Suppression-Induced Forgetting effect) of the TNT task. In the past, we offered a standardized on-site training in person (“traditional” approach), which included multiple sessions over a few days. The traditional training started with the trainee being the participant. We believe that experiencing the task directly (especially the challenge represented by the TNT phase) is essential in learning how to administer the TNT task properly. This is now achieved by watching the videos #1 (subjective experience) and #2 (interaction of experimenter and participant) provided in the TNT training package. Next, the trainee would receive instructions about how to launch the MATLAB functions and attend a lecture on becoming aware of various background methodological elements of the TNT task. This is now achieved by watching the videos #3 and #4. Subsequently, the trainee needed to run one or more participants, while being supervised by an experienced experimenter. We now offer the option of videoing oneself while administering the TNT task and sending it to us for feedback (cf. informed consent for video sessions in the TNT training package). Finally, after getting feedback and collecting data on a few participants (Module E), trainees attended a lecture from Dr. Anderson about potential pitfalls in running a TNT task, and suggested solutions (Section Practical guidelines). This is achieved by video #5. We believe that the availability of this material constitutes a substantial improvement in standardization. Obviously, we remain available for questions. The traditional and new steps are summed up in Table 2.

**Table 2:** Comparison between the former “traditional” approach to train prospective TNT experimenters, and the new training experience offered with the TNT training package (cf. ‘Guide to the TNT training.pdf’ and ‘TNT_training_intro.htm’ in the TNT training package)

Step #	Traditional approach	TNT training package	Training module
1	Take part in a TNT experiment as a participant	Video #1, DQ clips	A
2	Watch an experienced experimenter interact with a naïve participant	Video #2, DQ clips	A
3	Attend a lecture on the fundamentals of the TNT task	Video #4	A
4	Discuss with an experienced experimenter the TNT tasks (Q&A session)	HTML documentation	B
5	Study the TNT material and learn its structure and content	TNT material	C
6	Learn how to run the MATLAB functions	Video #3, MATLAB functions	A, D
7	Administer the TNT task to a naïve participant under supervision of an experienced experimenter	Video yourself and get feedback	E
8	Collect TNT data on a small group of participants (“flying solo”)	Administer the TNT task	E
9	Introduction to data analysis	Look at the TNT data	F
10	Meet with Dr. Anderson to reinforce key points	Video #5	A

#### Eye-tracking

Given its potential to monitor a key aspect of participants’ compliance (i.e., overt attention), if an eye-tracker is available, we recommend its adoption to make sure that participants attend the cues during the TNT phase. This way, non-attended trials can then be discarded from the analysis.

### Package limitations

Together with the present method paper, we provide a “TNT training package” which has been designed with the aim of standardizing and facilitating the training of prospective TNT experimenters. This was done to increase the reliability, replicability (Section Replicability of the Suppression-Induced Forgetting effect), and interpretability of TNT studies, as well as to enhance the communication and transferability of knowledge across different laboratories. However, like any other research tool, the current version of this training package has its limitations (listed below). Our plan is to keep updating this package over time, in order to fix its current limitations, and offer a research tool able to keep up with the various needs that will gradually arise in the field.

#### English language

Currently, the materials in the TNT training package are only available in English. This is not only true for the stimulus material, but also for all others (instructions, videos, questionnaires, FAQs, etc.). Indeed, our lab is in contact with a number of collaborators in other countries who developed (or are developing) their own stimulus material in different languages. Anyone who is willing to run a TNT study with verbal material in a languages other than English should feel free to contact us, to cross-check whether material in any required language is currently available and get access to such versions. Also, standardized, translated materials, are currently available upon request for 14 languages as a part of a multisite registered replication study (Fawcett et al., 2023; Section Words). As regards the rest of the package, we plan to deliver it in different languages in the future (to this aim, it would be helpful to reach out and express your interest, so that we can get an idea of how many researchers/laboratories in any given country may be interested, so that we may prioritize their language).

#### Verbal material

Similarly to the previous point, the current TNT training package only supports the use of verbal stimulus material (i.e., words), in spite of the fact that – at present – a good number of TNT studies have been using pictorial material. Of course, the implementation of pictorial material in the context of a TNT study would clearly be of interest and desirable (Section Pictures). However, we prioritized the delivery of a standardized (verbal) version of the package first, instead of delaying it any further in order to implement all possible nuances. After all, the MATLAB code we make available may be tweaked at will to accommodate various needs in a flexible manner. Anyway, we are currently working on a revised version of the MATLAB code and accompanying material, as well as on the selection of standardized pictorial material that will allow the implementation of visual stimuli in TNT studies.

#### Neutral material

A third limitation to the current version of the TNT training package is that its stimulus material is made up of only neutral material (i.e., words). Many TNT studies in recent years have manipulated emotional dimensions (e.g., valence and arousal) mainly using pictorial stimuli, showing that such dimensions modulate memory suppression (Section Emotional processing), especially in clinical populations. Again, we prioritized the opportunity to deliver a standardized procedure to the larger community over incorporating all possible nuances, and we are currently working to set up a version of stimulus material that includes emotionally valenced items.

#### Ceiling effects

Another limitation is the lingering issue of getting a ceiling effect while using the current verbal material (i.e., word pairs). In some studies, we have observed ceiling effects on the final recall test, especially on the same probe test. This may imply that some tuning of materials or presentation-rate parameters may be required in individual labs to ensure that final test performance is off ceiling, enabling SIF to be properly measured. In the case of clear ceiling effects on the SP test, focusing the analysis on unconditionalized data may be more appropriate.

#### Tailor-made feedback

Finally, we need to mention that currently there is no easy way for us to give feedback to individual research about the quality of their training. We have done our best to produce training materials that incorporate 20 years of experience, and that translate as closely as possible the experience offered by the “traditional” approach (i.e., inviting trainees to our lab for a whole week of training) into the new one offered by the TNT training package (cf. Table 2). In the latter, we offer trainees the opportunity to video themselves, send us the video, and obtain a tailor-made, detailed feedback about their performance as TNT experimenters. However, we acknowledge that, on the whole, the experience of self-administering the TNT training package is not fully equivalent to attending the TNT training in person. The latter approach used to offer a number of formal and informal opportunities for interaction with experienced TNT researchers which is hard to mimic in the standardized version. We are considering a number of strategies to improve the current TNT training package from this perspective, and we welcome any external input in this regard.

### Concluding remarks

In the present method paper, we have provided extensive details about the TNT task from a “behind the scenes” perspective. In doing so, we have reported the major steps in its development, a full description of its structure and materials, several issues that need to be carefully considered when planning a new study, a set of practical guidelines for good scientific practice in running TNT studies, and the basics of the analysis process. We have also provided standardized material to train prospective TNT experimenters. We believe that this paper and package will increase standardization, reliability, and replicability in the field of voluntary memory suppression across laboratories worldwide. Indeed, we are hopeful that the availability of a standardized procedure will have a huge positive impact on future investigations of memory control.

## Taking the training

In order to take the TNT training, please go to the following link: 10.5281/zenodo.7839893, download the *TNT training package* (“TNT_training_package.zip” file), and unzip it. This file is password-protected, and the password is: ***W3g98lQqk10j***. The file “Guide to the TNT training.pdf” contains a helpful overview of the training structure and training materials. Please read this document carefully before proceeding. Then, you can start your training from the file named “TNT_training_intro.htm” in the main folder. From there, you will be able to navigate the various training modules via a set of HTML pages, which will give you full access to all other multimedia files (i.e., tutorial videos, experimental materials, MATLAB functions, etc.). Please notice that the HTML pages have been optimized for Google Chrome.

## Data Availability

https://doi.org/10.5281/zenodo.7839893.

## Footnotes

1 The *Go/No-Go task* is a paradigm used to study response inhibition. It requires participants to respond by pressing a button when they see a “Go” signal, and not respond when they see a “No-Go” signal. The key behavior measured with this procedure is a participant’s ability to withhold a response on No-Go trials (i.e., a measure of their response inhibition ability).

2 *Associative interference* is the phenomenon whereby the retrieval of a given memory can be disrupted by the presence of other (currently undesired) traces related to the same memory.

3 *Effective connectivity* is a type of brain connectivity (i.e., an approach to investigating the brain in terms of how information flows between different regions within it). Effective connectivity is constrained by both anatomical connectivity (i.e., brain structure and physical connections between brain regions) and functional connectivity (i.e., brain regions which tend to co-activate concurrently, and therefore are supposed to jointly process some specific information), and account for causal relationships between regions (i.e., region A affects region B, and not vice-versa).

4 *Null events* are basically “fixation trials” whose length matches the duration of an actual stimulus. These are randomly interspersed with actual trials in order to optimize fMRI event-related designs, and allow a better estimation efficiency (i.e., response vs. inter-stimulus baseline). They are an easy way to randomize the stimulus-onset-asynchrony between actual events, and hence obtain what is called a “stochastic design” in fMRI.

## Code availability

https://doi.org/10.5281/zenodo.7839893.
